# Origin of High Efficiency and Long-Term Stability in Ionic Liquid Perovskite Photovoltaic

**DOI:** 10.34133/2020/2616345

**Published:** 2020-09-10

**Authors:** Lingfeng Chao, Tingting Niu, Hao Gu, Yingguo Yang, Qi Wei, Yingdong Xia, Wei Hui, Shouwei Zuo, Zhaohua Zhu, Chengjie Pei, Xiaodong Li, Jing Zhang, Junfeng Fang, Guichuan Xing, Hai Li, Xiao Huang, Xingyu Gao, Chenxin Ran, Lin Song, Li Fu, Yonghua Chen, Wei Huang

**Affiliations:** ^1^Frontiers Science Center for Flexible Electronics, Xi'an Institute of Flexible Electronics (IFE) and Xi'an Institute of Biomedical Materials & Engineering, Northwestern Polytechnical University, 127 West Youyi Road, Xi'an 710072, China; ^2^Key Laboratory of Flexible Electronics (KLOFE) & Institution of Advanced Materials (IAM), Nanjing Tech University (Nanjing Tech), Nanjing, 211816 Jiangsu, China; ^3^Shanghai Synchrotron Radiation Facility, Shanghai Institute of Applied Physics, Chinese Academy of Sciences, Shanghai 201204, China; ^4^Beijing Synchrotron Radiation Facility, Institute of High Energy Physics, Chinese Academy of Sciences, Beijing 100049, China; ^5^School of Physics and Electronic Science, Ministry of Education, Nanophotonics &Advanced Instrument Engineering Research Center, East China Normal University, Shanghai 200062, China; ^6^Institute of Applied Physics and Materials Engineering, University of Macau, Macau, Macao SAR 999078, China; ^7^Key Laboratory for Organic Electronics & Information Displays (KLOEID) and Institute of Advanced Materials (IAM), Nanjing University of Posts and Telecommunications, Nanjing, 210023 Jiangsu, China

## Abstract

Environment-friendly protic amine carboxylic acid ionic liquids (ILs) as solvents is a significant breakthrough with respect to traditional highly coordinating and toxic solvents in achieving efficient and stable perovskite solar cells (PSCs) with a simple one-step air processing and without an antisolvent treatment approach. However, it remains mysterious for the improved efficiency and stability of PSCs without any passivation strategy. Here, we unambiguously demonstrate that the three functions of solvents, additive, and passivation are present for protic amine carboxylic acid ILs. We found that the ILs have the capability to dissolve a series of perovskite precursors, induce oriented crystallization, and chemically passivate the grain boundaries. This is attributed to the unique molecular structure of ILs with carbonyl and amine groups, allowing for strong interaction with perovskite precursors by forming C=O…Pb chelate bonds and N-H…I hydrogen bonds in both solution and film. This finding is generic in nature with extension to a wide range of IL-based perovskite optoelectronics.

## 1. Introduction

Perovskite solar cells (PSCs) have attracted extensive attention in emerging photovoltaics with certified efficiency as high as 25.2% [1–4]. Compared to traditional photovoltaic technologies, such as silicon (Si) solar cells, gallium arsenide (GaAs) solar cells, and cadmium telluride (CdTe), one of the most attractive features of PSC is its solution-processable characteristic [5–8], which is able to remarkably reduce the cost of the process, and it is simple to implement the preparation of flexible devices [9–11]. It is worth noting that, currently, high-quality perovskite film preparation requires a complicated antisolvent method under an inert atmosphere based on *N*,*N*-dimethylformamide (DMF) and dimethyl sulfoxide (DMSO) as precursor solvents and chlorobenzene (CB) as an antisolvent [12–18]. In addition to solvent handling issues and toxicology concerns, antisolvent engineering with cumbersome processes requires a higher operating technique and environment, which seriously hinders the commercialization process of PSCs.

Recently, the breakthrough of solvent application in PSCs is employing novel protic amine carboxylic acid ionic liquids (ILs) as perovskite precursor solvents to replace traditional highly toxic and coordinating solvents [19–27], e.g., DMF and DMSO. ILs have the merits of environment-friendly, stable physical and chemical properties, low vapor pressure, wide electrochemical window, and high solubility [19]. The amine carboxylic acid IL methylammonium formate (MAFa) was first employed as a perovskite precursor solvent, and a large-area, orientationally pure crystalline MAPbI_3_ film with periodic microarrays and very low trap state density can be fabricated by a sharp liquid-to-solid transition [20]. At the same time, a new IL, methylammonium propionate (MAPa), was also demonstrated to be a novel solvent system for PSCs [22]. Recently, we successfully reported an IL solvent, methylammonium acetate (MAAc), which leads to power conversion efficiency (PCE) over 20% by a simple one-step method in humidity air without any additive [23]. Moreover, two-dimensional Ruddlesden–Popper PSCs employing IL solvents exhibited a certified PCE close to 18% with excellent stability [24]. Moreover, the Dion-Jacobson phase and tin-based PSCs based on ILs also showed excellent performance [26, 27], suggesting the universal characteristics of IL solvents. ILs are expected to be a promising solvent for the extensive adaptability in the field of perovskite optoelectronic technology and especially for industrial production requiring a simple and fast process. However, the lack of in-depth understanding of ILs on the origin of efficient and stable PSC is challenging the development of IL-based perovskite photovoltaic.

Here, we show that protic amine carboxylic acid ILs are able to combine the three functions of solvents, additive, and passivation into one system to achieve efficient and stable PSCs. Firstly, the ILs have the capability to dissolve a series of perovskite precursors by strong chemical interaction between the solvent and solute. Secondly, ILs can induce oriented crystallization and accelerate the conversion of precursor solution to perovskite crystal by forming active intermediates. Thirdly, the residual ILs *in situ* chemically passivate the grain boundaries by interacting with undercoordinated Pb and I. The unique molecular structure of protic amine carboxylic acid ILs is capable of improving optoelectronic properties and stability of the perovskite thin films and devices. We believe that the full understanding on ILs would enhance the pace of perovskite photovoltaic technology transition.

## 2. Results

### 2.1. Photovoltaic Device and Performance

**Figure 1:**
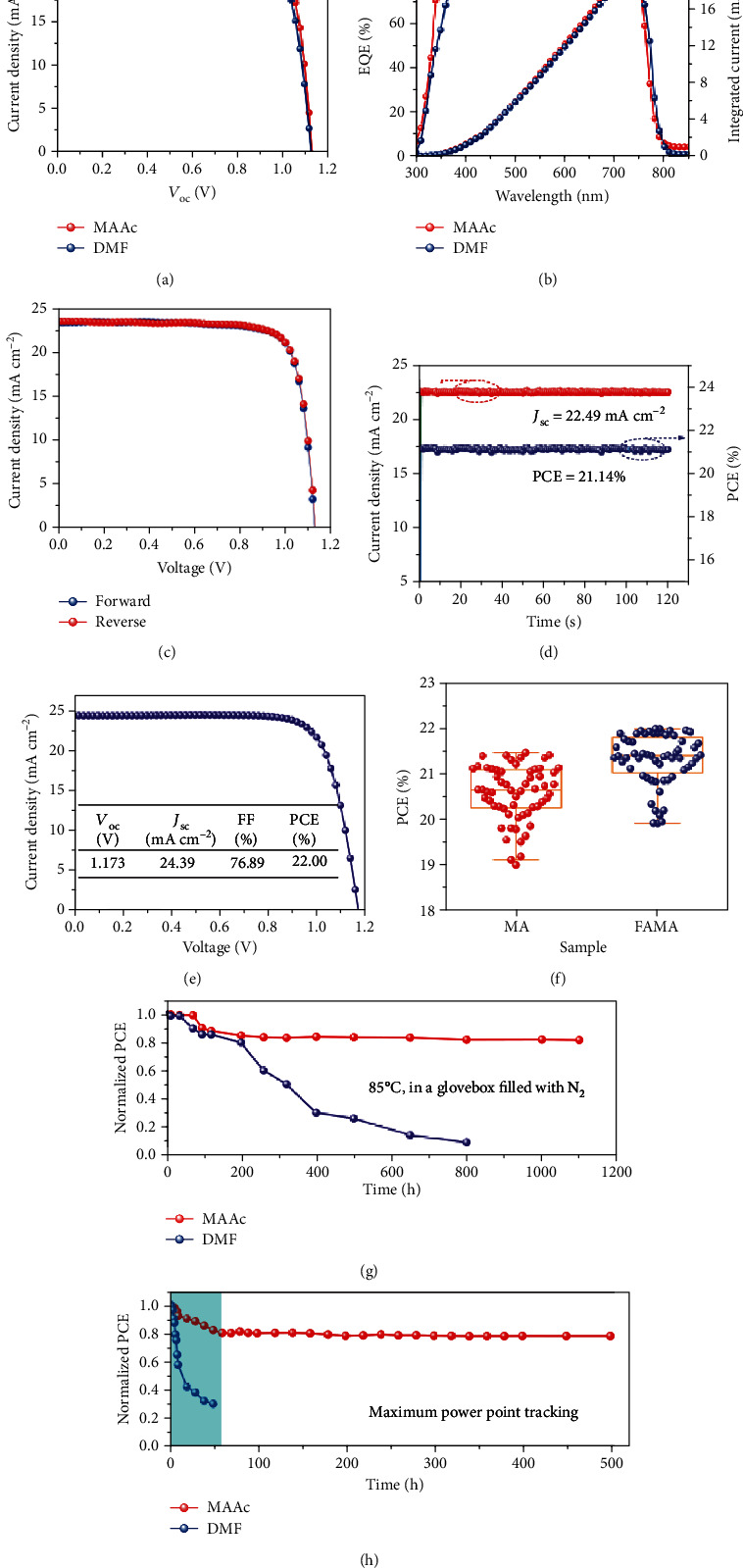
Photovoltaic performance and long-term stability of the PSCs. (a) The performance of the MAPbI_3_-based champion device. (b) EQE spectra and integrated *J*_SC_ of the MAPbI_3_-based champion device. (c) *J*-*V* curves under different scanning directions for MAAc PSCs. (d) The stabilized current density and power output of the MAPbI_3_ device for MAAc PSCs. (e) The performance of the champion device doped with FABr for MAAc PSCs. (f) The statistics of the PCEs of devices with and without FABr for MAAc PSCs. (g) Thermal stability of DMF and MAAc PSCs. The devices are maintained at 85°C condition in an N_2_-filled glovebox and measured regularly. (h) Operational stability of DMF and MAAc PSCs. MAAc and DMF PSCs are tracked at MPP (0.94 V and 0.90 V, respectively) under continuous 1-sun illumination in the glovebox.

Achieving efficient PSCs is an important prerequisite for evaluating preparation methods and studying the mechanism of film formation. We first fabricated the devices with a structure of indium tin oxide (ITO)/tin oxide (SnO_2_)/MAPbI_3_/2,2′,7,7'-tetrakis(*N*,*N*-di-methoxyphenylamine)-9,9′-spirobifluorene (Spiro-OMeTAD)/MoO_3_/Au. For the DMF solvent, high-quality film deposition processes require the use of antisolvent-assisted crystallization and need to be prepared in an inert atmosphere [12]. It should be noted that the thin films based on the MAAc solvent with large grains and low surface roughness (Figure S1) can be prepared in a natural environment with relative humidity of 20%~80% by a one-step approach without the use of antisolvent treatment (Figure S2). As a result, PCEs of 20.49% and 21.18% were achieved in the best-performing DMF- and MAAc-based PSCs (Figure 1(a)), respectively. The detailed performance parameters are shown in Table 1. As far as we know, the PCE of MAAc PSCs is one of the highest reported efficiencies based on MAPbI_3_ PSCs (Table S1). The external quantum efficiency (EQE) and integrated *J*_SC_ are shown in Figure 1(b), where the integrated *J*_SC_ of 22.01% and 22.60 mA cm^−2^, respectively, agrees well with the measured *J*_SC_ from a current-voltage (*J*-*V*) curve. Furthermore, MAAc devices show hysteresis-free *J*-*V* curves under a forward and reverse scan (Figure 1(c), Table S2), which may be due to the bulk and surface defects being passivated, inhibiting ion migration [10, 11]. As a result, the photocurrent density promptly achieves 22.49 mA cm^−2^ and a stabilized PCE of 21.14% at MPP (0.94 V) (Figure 1(d)). Furthermore, to further increase the PCE of the MAAc device, we doped FABr into MAPbI_3_. The PCE of 22% was achieved, which is the highest reported value in all one-step approaches in air (Figure 1(e), Table 1). Moreover, MAAc devices have high repeatability with the average efficiency of 20.58% (MAPbI_3_) and 21.33% (FABr-doped MAPbI_3_) (Figure 1(f)).

To further explore the effects of the two solvents on stability, the humidity, heat, and light stability of perovskite films and devices were measured. No change in XRD patterns was observed in MAAc perovskites, exposed in ambient air for even 2 years under humidity of 30%-80% (Figure S3). In addition, MAAc perovskite films can be maintained for 500 h and 800 h under 85°C and light conditions without the appearance of diffraction peaks of PbI_2_ (Figure S4). However, the DMF perovskite films suffer from serious degradation. Moreover, the maximum rate of MAAc perovskite powder decomposition is 351°C higher than that of DMF perovskite powder at 346°C with a thermal gravimetric analyzer (TGA) (Figure S5). Most importantly, the MAAc PSCs exhibited enhanced long-term stability, which retained more than 87% of their original PCE after 2000 hours in air (Figure S6), and the device after being placed in the air for 2500 h is shown in Figure S6, while the DMF devices maintain 30% of their original PCE merely after 1200 h and began to decay severely after 600 h (Figure S6), which may be attributed to the humidity effect with a large amount of generated PbI_2_, as demonstrated in Figure S4. Furthermore, the long-term stability of PSCs under continuous light stress for 1800 h was also studied (1 sun, in a glovebox filled with N_2_, Figure S7). The MAAc PSCs (more than 94% of the original PCE) exhibit obviously enhanced stability compared to DMF PSCs (around 73% of the original PCE). It is worth noting that MAAc PSCs can maintain more than 82% of the original efficiency for 1000 h at high temperature (85°C, in an N_2_-filled glovebox, Figure 1(g)) and have better thermal stability than DMF PSCs (around 10% of the original PCE for 800 h). Operational stability is the closest requirement to commercial photovoltaic applications. The stability of the device was traced for 500 h under MPP (0.94 V) (Figure 1(h)). The MAAc perovskite device rapidly decayed to the original 80% within 50 h and remained stable. However, the DMF device has been completely attenuated after 50 hours of continuous operation (0.90 V), showing extremely unstable performance.

The difference in efficiency and especially in stability of MAAc PSCs over DMF PSCs inspired us to reveal the origination. In the perovskite solution process, the solvent will participate in the entire process from solution to film. To comprehensively and deeply understand the influence of solvents on this process and the device, we conducted a detailed study from the precursor solution, the film formation process, the crystal structure, and the carrier transport characteristics of the device.

### 2.2. Chemical Interaction between the Solvent and Solute (Solvent)

**Figure 2:**
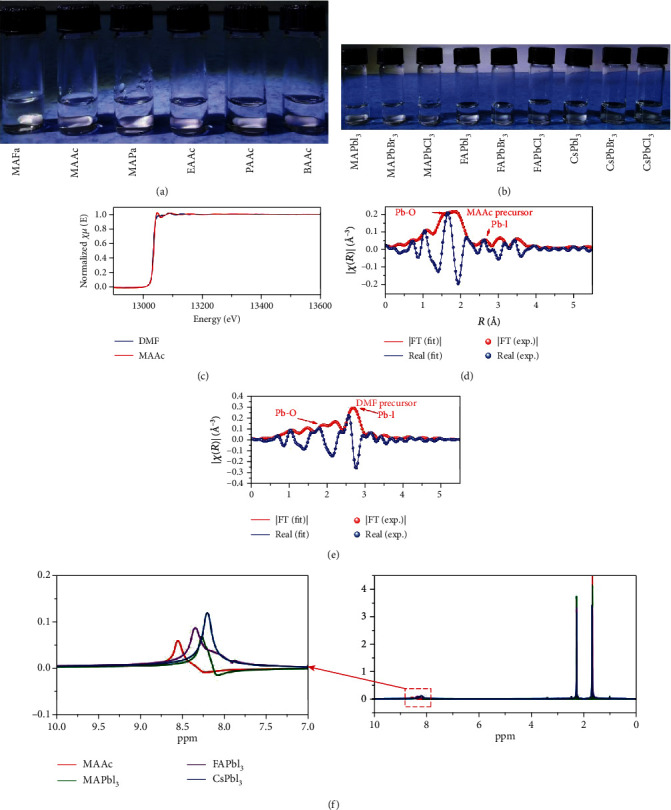
Perovskite precursor solution chemistry. (a) The solution images of the MAPbI_3_ perovskite precursor dissolved in different IL solvents. (b) The solution images of different perovskite precursors (MA-, FA-, and Cs-based perovskites) dissolved in the MAAc solvent. (c) The Pb *L*3-edge XANES spectra of the DMF and MAAc perovskite precursors. The *k*^2^-weighted Fourier transforms of the EXAFS spectra at the Pb *L*3-edge for the (d) MAAc and (e) DMF perovskite precursors. (f) ^1^H NMR spectra of a pure MAAc solvent and different perovskite precursors in the MAAc solvent.

Compared with traditional solvents, ILs have been widely used as solvents in industrial production because of their stable physical and chemical properties, negligible vapor pressure, and harmless properties [19]. Here, a series of novel amine carboxylic acid ILs, e.g., methylammonium formate (MAFa), methylammonium acetate (MAAc), methylammonium propionate (MAPa), ethylammonium acetate (EAAc), propylammonium acetate (PAAc), and butylammonium acetate (BAAc), were synthesized as solvents and used to prepare perovskite precursor solution. As shown in Figure 2(a), a hybrid perovskite MAPbI_3_ precursor (300 mM/ml) can be totally dissolved with colorless solutions. Moreover, different perovskite precursors containing MA-, FA-, and Cs-based perovskites can be effectively dissolved in the MAAc solvent as an example (Figure 2(b)), which strongly suggests a class of universal solvent for halide perovskites. Up to now, high-efficiency PSCs are prepared by perovskite precursor solution with mixed solvents, e.g., DMF and DMSO. Due to the differences in the physical and chemical properties of the solvents, however, a large number of defects are probably generated during the film formation process, which results in adverse impact on device performance. Amine carboxylic acid salt ILs as single solvents can effectively avoid the negative effects of solvent competition, which facilitates the preparation of high-quality films and high-efficiency PSCs.

The colorless characteristics indicate the similar interaction appearance in the amine carboxylic acid IL solutions. For better understanding the interaction between ILs and perovskite precursors, we take MAAc as an example to discuss. We first studied the chemical bonding environment of Pb atoms based on DMF and MAAc solvents by the X-ray absorption near-edge structure (XANES). The MAAc precursor solution XANES data exhibit a strong shoulder peak at approximately 13050 eV than those from the DMF precursor solution (Figure 2(c)), which was attributed to the presence of Pb-O coordination from the coordination complex of PbI_2_-MAAc, as in previous reports [7, 28, 29]. We therefore further performed a linear combination fit for the real-space extended X-ray absorption fine structure (EXAFS) spectra of MAAc and DMF precursor solutions, as shown in Figures 2(d) and 2(e). A strong Pb-O coordination peak was observed at near 1.65 Å in the MAAc precursor solution, and a small amount of Pb-I coordination is present at near 2.6 Å. In contrast, DMF precursor solution exhibited a strong Pb-I coordination peak with a small amount of Pb-O coordination, which indicates that the perovskite precursor in the DMF solvent is dominated by iodine complexes due to the greater coordination capacity of I^−^ than DMF, as in previous reports [29, 30]. Moreover, as shown in Figure 2(f), the ^1^H NMR spectra of the three perovskite precursor solutions (MAPbI_3_, FAPbI_3_, and CsPbI_3_) showed the clear blue shift of the N-H peak with respect to the pure MAAc solvent (shown in an internal enlarged view in Figure 2(f)), which proved the formation of hydrogen bonding of N-H⋯I between MAAc and PbI_2_ and/or MAI (CsI). Therefore, it is now clear that MAAc dissolves the perovskite precursors by forming Pb-O coordination and N-H⋯I hydrogen bonds, which is different from the traditional iodoplumbate complexes [18]. The complicated chemical bond system consisting of strong chelation and hydrogen bonding interaction provides a necessary encapsulation of the perovskite precursor, leading to the colorless solution and making the precursor solution stable. Based on the unique dissolution characteristics of MAAc over traditional solvents, e.g., DMF and DMSO, the saturation concentration of the perovskite precursor solution can reach 4 M, which far exceeds those of DMF (1.5 M), DMSO (1.9 M), and GBL (2.2 M) [31]. Higher saturation concentration has the benefit to allow the fabrication of thick perovskite films, which has been demonstrated to significantly improve the stability of PSCs [32].

### 2.3. Perovskite Crystallization and Structure Transformation (Additive)

**Figure 3:**
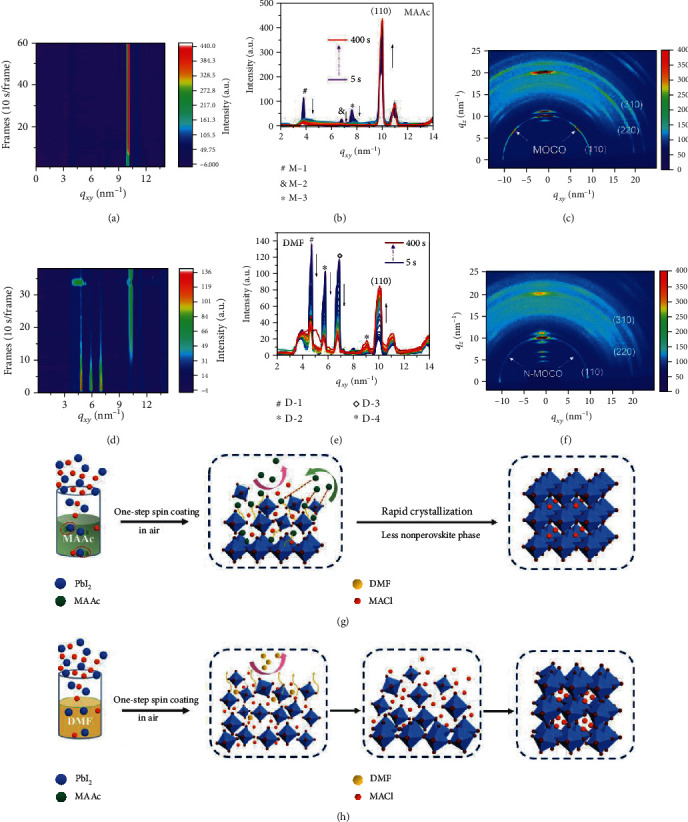
The crystallization and structure transformation by the time-resolved GIWAXS. (a) 2D mapping of a real-time evolution process based on MAAc perovskites. (b) 1D GIWAXS diffraction pattern of MAAc thin films in the *q*_*xy*_ direction for 40 frames (10 s per frame). (c) 2D GIWAXS diffraction images of MAAc perovskites. (d) 2D mapping of a real-time evolution process based on DMF perovskite. (e) 1D GIWAXS diffraction pattern of DMF thin films in the *q*_*xy*_ direction for 40 frames (10 s per frame). (f) 2D GIWAXS diffraction images of MAAc perovskites. The diagram of the mechanism of the perovskite crystal growth process in different solvents: (g) MAAc and (h) DMF.

Solvents can affect the chemical properties of the precursor solution and determine the crystallization of perovskite. To understand the effect of the MAAc solvent on perovskite crystallization, we first investigate the entire crystal growth dynamic process by *in situ* real-time grazing incidence wide-angle X-ray scattering (GIWAXS), as compared to that in the traditional DMF solvent (the scheme diagram of the GIWAXS setup is shown in Figure S8). Note that the MAAc perovskite precursor solution is firstly spin coated onto an ITO/SnO_2_ substrate at room temperature. A transparent oily-like noncrystalline thin film is obtained (Figure S9), which indicates a highly concentrated perovskite precursor. Then, the films are continuously annealed at 100°C in ambient air and radiated by X-ray at the same time. The false color intensity maps in Figures 3(a) and 3(d) show the evolution of the perovskite crystal structure over time based on MAAc and DMF solvents, respectively. We found that the (110) crystal plane (*q*_*xy*_ = 10 nm^−1^) of the MAAc perovskite film reaches the strongest crystalline state much faster than that of the DMF perovskite film. The position of the (110) crystal plane in the MAAc perovskite film takes only 70 s to fully convert to *q*_*xy*_ = 10 nm^−1^, while the DMF perovskite film needs 130 s (Figure S10), which corresponds to the rapid crystallization phenomena of the MAAc perovskite film in Figure 3(a). Moreover, the MAAc perovskite film exhibited a larger peak area and narrower diffraction full width at half maximum (FWHM) of the (110) crystal plane than the DMF perovskite film (Figure S11). It should be noted that no changes on the peak area and FWHM of the MAAc perovskite film over time were observed (Figures S11 and S12), corresponding to the steady crystalline state of the MAAc perovskite film over that of the DMF perovskite film in Figure 3(a). From the intensity distribution curve of *in situ* 1D GIWAXS integral (Figure 3(b)), nonperovskite diffraction peaks were observed at M-1 (*q*_*xy*_ = 3.8 nm^−1^), M-2 (*q*_*xy*_ = 6.8 nm^−1^), and M-3 (*q*_*xy*_ = 7.6 nm^−1^) in the MAAc perovskite film at the beginning of annealing. They are weaker with respect to the perovskite phase (*q*_*xy*_ = 10 nm^−1^), and the diffraction intensity rapidly weakens or even disappears with increasing annealing time. This is because the precursor has formed a dispersed uniform cluster in the MAAc system due to the strong component interaction, which inhibits the formation of the nonperovskite phase and promotes the quick transformation of the stable perovskite phase. However, a large number of nonperovskite phases were observed for the DMF perovskite film located at *q*_*xy*_ = 3.8 nm^−1^ (D-1), *q*_*xy*_ = 4.7 nm^−1^ (D-2), *q*_*xy*_ = 6.9 nm^−1^ (D-3), and *q*_*xy*_ = 8.0 nm^−1^ (D-4), which did even not disappear with the growth of perovskite crystals (Figure 3(e)).

To further study the crystal growth and orientation, we extracted 2D GIWAXS diffraction images of the final perovskite films. A series of similar bright diffraction patterns were obtained at *q*_*xy*_ = 10, 20, and 22.4 nm^−1^, corresponding to the (110), (220), and (310) crystal planes, respectively (Figures 3(c) and 3(f)). It can be seen that the DMF thin film exhibits multiple diffraction rings (Figure 3(f)), indicating that the crystals are randomly arranged, which may result in a large number of defects [33]. In contrast, discrete Bragg spots can be clearly observed on the diffraction ring of each crystal plane in the MAAc perovskite film (Figure 3(c)), which indicates that the perovskite crystals are ordered in multiple directions and have a better preferred out-of-plane orientation [34]. In order to further quantitatively analyze the multiple and ordered crystal orientations (MOCO) of perovskite grains, we obtained the corresponding azimuthal distribution map by radial integration of the (110) diffraction rings of the two films (Figure S13). The MAAc perovskite film exhibits a narrower sharp peak at the azimuth angle of 90°, indicating that the perovskite film has a higher degree of orientation. Meantime, the azimuth angles of 21.8° (180°-21.8°) and 45.0° (180°-45.0°) were also observed in the MAAc perovskite film, which also represents the preferential orientations of the crystal. In addition, the azimuthal distribution of time evolution also proves that MAAc can quickly crystallize and stabilize rapidly (Figure S14). These results demonstrate that MAAc can promote the ordered and highly oriented growth of perovskite in multiple directions, which greatly enhances the extraction and transport of carriers of perovskites, thereby improving the device performance of the PSCs [34].

These results show that MAAc is not only a good solvent but also an effective additive for perovskite crystallization. Figures 3(g) and 3(h) present the schematic of the evolution process of perovskite films from precursor solution to high-quality crystals. For precursor solution, as shown by equation (1), when the PbI_2_ and MAl were dissolved in MAAc, Pb^2+^ will combine with CH_3_COO^−^ (Ac^−^) to form a weak electrolyte Pb(Ac)_2_. Furthermore, Pb^2+^ will also combine with the surrounding MAI to form agglomerates with less binding energy. The thermodynamic instability of Pb(Ac)_2_ causes the precursor to evaporate CH_3_COOH (HAc) and CH_3_NH_2_ (MA) gases upon heat treatment, prompting the further rightward reaction of the following equation (2).

(1) $$\begin{array}{c} {\text{PbI}}_{2}+\text{C}{\text{H}}_{3}{\text{NH}}_{3}\text{I}+\text{C}{\text{H}}_{3}{\text{COONH}}_{3}{\text{CH}}_{3}⟶\text{Pb}{\left({\text{CH}}_{3}\text{COO}\right)}_{2}\cdots {\text{CH}}_{3}{\text{NH}}_{3}\text{I} \end{array}$$

(2) $$\begin{array}{c} \text{Pb}{\left({\text{CH}}_{3}\text{COO}\right)}_{2}\cdots {\text{CH}}_{3}{\text{NH}}_{3}\text{I}⟶{\text{CH}}_{3}{\text{NH}}_{3}{\text{PbI}}_{3}+\text{C}{\text{H}}_{3}\text{COOH}↑+\text{C}{\text{H}}_{3}{\text{NH}}_{2}↑ \end{array}$$

Compared to DMF, MAAc as a solvent provides the above reaction process inside the precursor solution, avoiding the formation of multiple intermediate phases during the perovskite film crystallization process, which directly blocks the channel of defect generated. Notably, the agglomerates in equation (2) may be thermodynamically unstable coordination complexes between CH_3_COO^−^, Pb^2+^, and MA^+^, which may explain the change of peak position at the beginning of the annealing in Supplementary Fig. 10. Due to the fact that Pb^2+^ is combined with Ac^−^ in the form of a covalent bond, it will evaporate in the fixed direction of the covalent bond force rather than in all directions under the heat treatment. At the same time, I^−^ could quickly migrate to the crystal lattice to replace Ac^−^. As a result, the perovskite grows orderly along the direction of the force (Figure 3(g)). However, for the traditional solvent DMF, the crystallization process must first evaporate the solvent; then, the crystals are randomly arranged, and finally a perovskite structure can be achieved (Figure 3(h)).

### 2.4. *In Situ* Chemically Anchored Grain Boundaries (Passivation)

**Figure 4:**
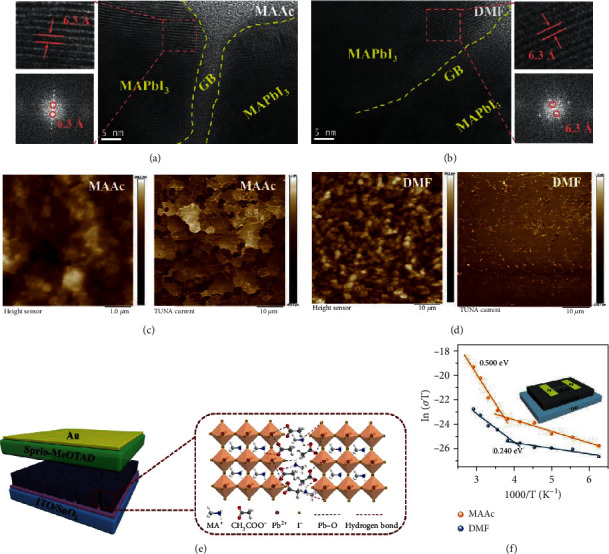
Microstructure characterization of perovskites. High-resolution transmission electron microscopy (HR-TEM) images of (a) MAAc perovskite (left: the magnified lattice fringes and FFT about the red frame region) and (b) DMF perovskite (right: the magnified lattice fringes and FFT about the red frame region). Conductive atomic force microscopy (c-AFM) and topological images of (c) MAAc perovskite and (d) DMF perovskite. (e) The structure diagram of grains and GBs in the MAAc perovskite. (f) The temperature-dependent conductivity of MAAc and DMF perovskite films for the activation energy (*E*_a_) of ion migration.

The features of high boiling point and low vapor pressure of MAAc determine that it may not be completely evaporated in the final perovskite film. As shown in Figure S15, the C=O (1600.68 cm^−1^) stretching vibration from Ac^−^ can still be clearly observed in the MAAc perovskite powder, which confirms the residue of MAAc in the final perovskite film. Considering the above GIWAXS patterns, we have not found an obvious shift in the diffraction angle in the DMF and MAAc perovskite films, which suggests that residual MAAc does not enter the lattice. Therefore, we speculate that MAAc may exist at the grain boundaries (GBs) of the perovskite films. To confirm our assumption, we used high-resolution transmission electron microscopy (HR-TEM) to examine the detailed structure information of grains and GBs in the DMF and MAAc perovskites. The clear amorphous GB walls were observed in the MAAc perovskite film (Figure 4(a)), while no obvious amorphous region was observed at GBs in the DMF perovskite film (Figure 4(b)), which confirms the presence of MAAc at GBs [28, 35]. Moreover, the red frame region was magnified and analyzed using fast Fourier transform (FFT), in which an interplanar spacing of 6.3 Å completely coincides with the (110) planes of MAPbI_3_ [36]. We found the same lattice fringes and FFT image in the DMF perovskite film, demonstrating the same perovskite structure in both films, corresponding to the GIWAXS patterns. To assess the passivation effect, the local conductivity of the MAAc and DMF perovskite films was measured by conductive atomic force microscopy (c-AFM) under a bias voltage of 2 V (Figures 4(c) and 4(d)). The brighter contrast of the MAAc and DMF film current images (average photocurrent 1.27 nA vs. 0.45 nA) indicated more conducting current flow through the MAAc perovskite film, which suggests that the MAAc perovskite film is beneficial to carrier separation and improves the photovoltaic performance [37]. Moreover, GBs of the MAAc perovskite film show brighter contrast, indicating that the GBs can efficiently flow the current in the MAAc perovskite film. In contrast, the obvious current contrast was not observed between grains and GBs in the DMF perovskite film, and we only can see uneven and discontinuous bright spots. This strongly demonstrated that the GBs for the MAAc perovskite film can clearly be considered charge transporting channels and further confirm the passivation effect of MAAc in the perovskite film [7, 28].

We further explored the passivation mechanism of MAAc at the GBs. We dissolved PbI_2_ into the MAAc solvent and investigated the characteristics of powders obtained from the PbI_2_-MAAc films. As shown in Figure S15, the C=O vibration peak (1604.51 cm^−1^) in the PbI_2_-MAAc powder shifted to a low wavenumber as compared to C=O (1645.23 cm^−1^) in the pure MAAc, which is attributed to the coordination interaction between C=O of MAAc and undercoordinated Pb^2+^ (I^−^ vacancy) in the PbI_2_-MAAc films [38]. Moreover, we found that the position of Pb^2+^ (4f_5/2_, 4f_7/2_) in the PbI_2_-MAAc film has tendency towards lower binding energy relative to the PbI_2_-DMF film in the X-ray photoelectron spectroscopy (XPS) spectra (Figure S16). The peak of the Pb-O bond can further be observed in Raman spectrum of the PbI_2_-MAAc film (Figure S17). These results confirm the strong interaction between undercoordinated Pb^2+^ and O atom in Ac^−^, which are consistent with previous reports that the Lewis base site C=O group can provide an electron pair to the undercoordinated Pb^2+^ in the perovskite, thereby effectively passivating the perovskite film [7, 38]. Together with the hydrogen bonds, therefore, the diagram of grains and GBs of MAAc perovskites can be drawn in Figure 4(e), in which the MAAc at GBs stabilized by intrinsic hydrogen bonding between MA^+^ and Ac^−^ can anchor perovskite octahedron through Pb-O (undercoordinated Pb^2+^ of MAPbI_3_ and C=O of MAAc), N-H⋯I (MA^+^ of MAAc and I^−^ of MAPbI_3_), and N-H⋯O (MA^+^ of MAPbI_3_ and C-O of MAAc), which can effectively stabilize the structure of perovskite and inhibit defects [7, 28].

To examine the effect of passivation of MAAc, we explored the trap density, ion migration, and carrier transport kinetics in the final MAAc perovskite films in comparison to the DMF perovskite films. For trap density, the bulk trap density of the DMF perovskite film is calculated to be 5.17 × 10^16^ cm^−3^ by femtosecond transient absorption (fs-TA) spectra, respectively (Figure S18). In sharp contrast, the bulk trap density of the MAAc perovskite film is an order of magnitude lower than that of the DMF perovskite film, which is only 3.90 × 10^15^ cm^−3^ (Figure S18). The lower defect state density of the MAAc perovskite was further confirmed by capacitance-voltage (*C*-*V*) measurement (3.16 × 10^15^ cm^−3^) (Figure S19). Furthermore, the dark current density of the MAAc device is obviously lower than that of the DMF device, indicating that MAAc perovskite could facilitate the charge transport and reduce the charge recombination (Figure S20) [23]. Compared to the DMF devices, the MAAc device has a smaller capacitance in the low-frequency region compared to the DMF device, which again proves that the MAAc device has fewer defects and less recombination (Figure S21) [23].

For ion migration, the lateral device structure is used to measure ion migration activation energy (*E*_a_) of ion conduction (Figure 4(f)), which represents the ease of ion migration, and can be obtained from the temperature dependence of the conductivity in the perovskite film [28]. The ion migration rate in perovskite is calculated by *E*_a_ according to the Nernst–Einstein equation:

(3) $$\begin{array}{c} \sigma \left(T\right)=\frac{\sigma_{0}}{T}\exp\left(\frac{E_{\text{a}}}{kT}\right), \end{array}$$

where *k* represents the Boltzmann constant and *σ*_0_ is a constant. *E*_a_ is obtained from the slope of ln (*σT*)–(1/*kT*) [39]. As shown in Figure 4(f), ions start to migrate at 268 K in the MAAc perovskite film, while the threshold temperature drops to 248 K in the DMF perovskite film. Meanwhile, *E*_a_ in the MAAc perovskite film is significantly increased from 0.24 eV (DMF perovskite film) to 0.48 eV, which indicates that ion migration is effectively suppressed in the MAAc perovskite films compared to the DMF perovskite films. The reduction of defects and the suppression of ion migration are attributed to the passivation of GBs by residual MAAc in the film. Perovskite crystals with low defects and suppressed ion migration are beneficial for long-term stability of films and devices [40–42].

For carrier transport kinetics, we monitored the kinetic traces at a band edge (PB2) of the MAAc and DMF perovskite films by the TA spectra (Figures S22a and S22d). The DMF perovskite film has an average lifetime of only 8.1 ns, while the MAAc perovskite film has a prolonged average lifetime of 78.6 ns due to the lower trap density (Figures S23a and S23b). In regard to the carrier lifetimes at the band edge of perovskite, they were substantially shortened when the perovskites were interfaced with SnO_2_ (Figures S22b and S22e) and Spiro-OMeTAD layers (Figures S22c and S22f), with fitted lifetimes of 23.2 and 8.5 ns for the MAAc perovskite film (Figure S23a), as compared to 3.2 ns and 3.7 ns for the DMF perovskite film, respectively (Figure S23b). The charge carrier transfer efficiency can be estimated to be 70% for MAAc perovskite/Spiro-OMeTAD and 90% for SnO_2_/MAAc perovskite, while DMF perovskites gave those even as low as 54% and 60% (Tables S3 and S4). In addition, the electron and hole diffusion lengths (*L*_D_) perpendicular to the film surface were calculated using the diffusion model. The MAAc perovskite gave longer electron and hole *L*_D_ of 138 nm and 257 nm, respectively, than those of 111 nm and 98 nm, respectively, for the DMF perovskite.

## 3. Discussion

In summary, we have revealed the origin of high efficiency and long-term stability in protic amine carboxylic acid IL-based perovskite photovoltaic. We concluded that ILs have the “three-in-one” functions of solvents, additive, and passivation for facial fabrication of efficient and stable PSCs. The unique chemical bonding properties of ILs by chelation and hydrogen bonding allow the dissolution of perovskite precursors. The interaction further enables the fast transformation of the precursor solution to a high-purity perovskite phase. Most importantly, the residual MAAc can *in situ* anchor the GB and effectively passivate the defect. The passivation mitigates interfacial voids and trap sites which aid in ionic migration, thus avoiding hysteresis behavior. We think that symptomatically designed ILs with different functions can accelerate the large-scale conversion of perovskite photovoltaics.

## 4. Materials and Methods

### 4.1. Materials

Lead iodide (PbI_2_, 99.99%) was purchased from TCI. Spiro-OMeTAD was purchased from Youxuan Tech, China. Methylamine hydrochloride (MACl), methylammonium iodide (MAI), caesium iodide (CsI, 99.999%), formamidinium iodide (FAI), and methylammonium bromide (MABr) were purchased from Greatcell Solar. Chlorobenzene (CB, anhydrous), *N*,*N*-dimethylformamide (DMF, anhydrous), methylamine solution (MA, 33% in H_2_O), ethylic acid (HAc, 99%), butylamine hydrochloride (BACl, 99.99%), ethylamine solution (EA), butylamine solution (BA), propylamine, formic acid, propanoic acid, and n-butyric acid were purchased from Sigma-Aldrich. All the materials were used without further purification.

### 4.2. Synthesis of Ionic Liquids (ILs)

MAFa, MAAc, MAPa, and MABa were synthesized by reaction of methylamine with the corresponding carboxylic acid. EAAc, BAAc, and PAAc were synthesized by reaction of acetic acid with the corresponding amine.

#### 4.2.1. MAFa

At -16°C, 15 ml of formic acid was first diluted with 62.5 ml of anhydrous methanol, followed by slowly adding it to a round-bottom flask containing 62.5 ml of methylamine (33% ethanol solution) and 25 ml of absolute ethanol, which was stirred for 2 h. The excess solvent is evaporated away with a rotary evaporator at 55°C to obtain the product MAFa.

#### 4.2.2. MAAc

Acetic acid (glacial, 30.6 ml, 0.654 mol, Fisher Scientific) and methylamine (55.6 ml, 0.982 mol, 40% in ethanol, Aldrich) were stirred in a 500 ml round-bottom flask for 2 h (ice bath) [23]. Afterwards, the liquid chemical MAAc was obtained by rotary evaporation at 80°C for 1 hour. The liquid product was put into a refrigerator for 2 hours to crystallize. The crystallized product was washed 3-5 times with ether for further purification; then, it was dissolved in ethanol and the excess liquid was distilled off with a rotary evaporator to obtain an ionic liquid product at 80°C for 1 h. Finally, the liquid product was cooled down to room temperature before use. MAPa, MABa, EAAc, BAAc, and PAAc have the same synthesis method.

### 4.3. Preparation of MAAc Precursor Solution

PbI_2_ and MAI were mixed in MAAc (400 mg/ml) or DMF (400 mg/ml) by reaction at 60°C for 6 hours to obtain perovskite precursor solution. The molar ratio for PbI_2_ : MAI is 1 : 1. And PbI_2_, MAI, and FABr were mixed in MAAc (400 mg/ml) by reaction at 60°C for 12 hours to get mixed cationic precursor solution. The molar ratio for PbI_2_ : MAI : FABr is 5 : 4 : 1.

### 4.4. Device Fabrication

ITO glass substrates were cleaned with detergent, deionized water, acetone, and isopropanol, in sequence, then dried in an oven at 120°C for 30 minutes, and treated with ultraviolet ozone for 15 minutes. SnO_2_ was deposited on an ITO substrate at 3000 rpm for 30 s and then annealed at 150°C for 30 minutes. Perovskite precursor solution was deposited on the SnO_2_ substrate at 4000 rpm for 20 s at a constant temperature under ambient conditions. After annealing for 5 min, Spiro-OMeTAD in CB solution (72.6 mg ml^−1^) with 17.5 *μ*l of lithium bistrifluoromethylsulfonate (LiTFSI) and 29 *μ*l of 4-*tert*-butylpyridine was deposited on the perovskite layer by spin coating at 4000 rpm for 30 s. Finally, MoO_3_ (5 nm) and Au (100 nm) were sequentially deposited on the hole transport layer (vacuum is 1.1 × 10^−4^ Pa).

### 4.5. Device Characterization

All the devices were tested in an N_2_-filled glovebox using a Keithley 2400 source meter under a simulated AM1.5G spectrum and a solar simulator (Enli Tech, Taiwan). The light intensity was calibrated to be 100 mW cm^−2^ using a NIST-certified monocrystalline Si solar cell (Newport 532 ISO1599). The device performance parameters were calculated from the current-voltage curves of the photovoltaic device under illumination. No protocol for preconditioning the device before its characterization was followed. The active area of the device is 0.05 cm^2^, and a black mask of 2.5 × 2 mm^2^ is used to fix the effective area of the device during the test. The external quantum efficiency of the device is characterized by the EQE measurement system (Enli Tech).

### 4.6. Lifetime Characterization

(1) For humidity stability characterization, the perovskite films or devices were placed in ambient air and 30%-80% relative humidity at room temperature. (2) For thermal stability characterization, perovskite films or devices are continuously heated at a temperature of 85°C in an N_2_-filled glovebox. (3) For light soaking stability characterization, the light intensity was 1-sun AM1.5G illumination, and the device temperature was kept at room temperature (25°C). The operational stability of devices was characterized at the MPP through a sunlight simulator with AM1.5G light in an inert gas glovebox for 500 h.

### 4.7. Characterization

XPS was measured with a PHI 5000 VersaProbe III with a microfocused scanning Al K*α* X-ray source. The S *K*-edge (2472 eV) XAFS spectrum of the sample was measured at the beamline (4B7A) of the Beijing Synchrotron Radiation Facility (BSRF), Institute of High Energy Physics (IHEP), Chinese Academy of Sciences (CAS). Atomic force microscope images including morphology and conductivity were obtained using a Park XE7 in a noncontact mode. SEM images of the samples were taken by Hitachi S-3400N SEM. The XRD of the sample was characterized by a Smart Lab diffractometer from Japan. The Fourier transform infrared spectroscopy (FTIR) of the sample is characterized by an IR spectrometer instrument (Thermo, Nicolet 6700). The sample style is powder. ^1^H NMR (600 MHz) was characterized in deuterated DMSO-*d*_6_. Transmission electron microscopy (TEM) images were obtained with JEOL2100 from Japan. The capacitance-voltage (*C*-*V*) spectrum was recorded with a Zahner Zennium electrochemical workstation at a frequency of 1 kHz for extracting *V*_b_. TGA spectra were obtained using the thermogravimetric analyzer TA Q500 with a flow rate of 20 ml min^−1^. The temperature is set from 25 to 800°C at a rate of 10°C min^−1^.

### 4.8. Grazing Incidence Wide-Angle X-Ray Scattering (GIWAXS)

GIWAXS was characterized at the beamline BL14B1 of the Shanghai Synchrotron Radiation Facility (SSRF). The sample was tested with an X-ray beam of *λ* = 0.68877 Å at an incident angle of 0.20° for 400 s, and the scattered light was detected with MarCCD at a distance ~746 mm vertically from the sample. The MAAc and DMF perovskite precursor solution is firstly spin coated on a Si/SnO_2_ substrate at room temperature, respectively. An oily-like noncrystalline perovskite film is obtained after spin coating. Then, the film is placed on a room temperature sample stage, and the GIWAXS measurement is triggered and the temperature of the sample stage is raised simultaneously.

### 4.9. X-Ray Absorption Spectroscopy (XAS) Measurements

The XAFS spectra of the Pb L3 edge (13035 eV) for precursor solution were characterized at the 1W1B beamline of the Beijing Synchrotron Radiation Facility (BSRF). It is provided with a double Si (111) monochromator which was used to monochromatize the X-ray white beam. Spectral data were processed and analyzed using Athena [Ravel, B., Newville, M., 2005. ATHENA, ARTEMIS, HEPHAESTUS: data analysis for Xray absorption spectroscopy using IFEFFIT. J. Synchrotron Radiat. 12, 537e541.].

### 4.10. Transient Absorption Spectroscopy

Transient absorption (TA) properties of the film with different functional layers were measured using a Helios setup, where a nondegenerate pump-probe configuration was applied to probe the transient dynamics in a femtosecond to nanosecond time region (50 fs-7 ns). An optical parametric amplifier (OPerA Solo), pumped by a 1 kHz regenerative amplifier (Coherent Libra, 800 nm, 50 fs, 4 mJ), was used to generate pump pulses. To seed the amplifier, a mode-locked Ti-sapphire oscillator (Coherent Vitesse, 80 MHz) was used. The laser from the regenerative amplifier was directly used for the pump at 800 nm, while a beta barium borate (BBO) crystal was used to double the frequency of 800 nm for the pump at 400 nm. White light continuum was applied as the probe pulses, which were formed by 800 nm femtosecond pulses via a sapphire plate (2 mm) for the 400-800 nm region.

### 4.11. Activation Energy Measurement of Ion Migration

The sample was measured on a copper substrate with temperature control by a heater and injected liquid He in the Lakeshore Probe Station under vacuum (1.1 × 10^−4^ Pa). The current was detected with Keithley 4200-SCS at 150 s after the voltage is switched on. It is worth noting that the device is first cooled to room temperature and then programmed to a predetermined temperature.

## Figures and Tables

**Table 1:** Summary of PSC parameters of different systems.

Solvents	*V*_OC_ (V)	*J*_SC_ (mA cm^−2^)	FF (%)	PCE (%)
DMF (MA)	1.129 (1.107 ± 0.02)	22.97 (22.87 ± 0.13)	78.67 (76.94 ± 1.68)	20.49 (19.48 ± 0.98)
MAAc (MA)	1.132 (1.117 ± 0.02)	23.39 (23.33 ± 0.07)	80.01 (78.96 ± 1.03)	21.18 (20.58 ± 0.61)
MAAc (MA/FA)	1.173 (1.164 ± 0.01)	24.39 (24.28 ± 0.35)	76.87 (75.47 ± 1.21)	22.00 (21.33 ± 0.57)

## Supplementary Materials

Supplementary Materials Figure S1: morphology of thin films. Figure S2: scheme image of preparation of the perovskite film. Figure S3: long-term stability of MAAc perovskite films in ambient air for even 2 years under humidity of 30%-80%. Figure S4: thermal (85°C) and light (1-sun AM1.5G illumination) stability of MAAc and DMF perovskite films in an N_2_-filled glovebox. Figure S5: TGA spectra of MAAc and DMF perovskite powder. Figure S6: air stability of PSCs. Figure S7: light soaking stability of PSCs. Figure S8: the experimental setup for in situ GIWAXS characterization. Figure S9: a transparent oily-like noncrystalline MAAc perovskite film. Figure S10: time evolutions of peak positions for the (110) crystal plane. Figure S11: time evolutions of the full width at half maximum (FWHM) for the (110) crystal plane through multipeak mode fitting. Figure S12: time evolutions of peak areas for the (110) crystal plane through multipeak mode fitting. Figure S13: the azimuthal distribution mapping by radial integration of the (110) diffraction rings of MAAc and DMF perovskite films. Figure S14: the azimuthal distribution mapping by radial integration of the (110) diffraction rings of time evolution of MAAc and DMF perovskite films. Figure S15: FTIR spectra of MAAc-based perovskite powder, MAAc+PbI2 powder from PbI2@MAAc solution, and pure MAAc solvent. Figure S16: the X-ray photoelectron spectroscopy (XPS) spectra of Pb (4f) of MAAc- and DMF-based PbI2 films. Figure S17: Raman spectra of the MAAc-based PbI2 film. Figure S18: the bulk trap state density of MAAc and DMF perovskite films calculated from TA spectra. Figure S19: defect state density of MAAc perovskite, measured by capacitance-voltage (*C*-*V*) with the device structure of ITO/SnO_2_/perovskite/Spiro-OMeTAD/MoO_3_/Au. Figure S20: the dark current density of the MAAc and DMF perovskite devices with the structure of ITO/SnO_2_/perovskite/Spiro-OMeTAD/MoO_3_/Au. Figure S21: admittance spectra of MAAc and DMF perovskite devices with the structure of ITO/SnO_2_/perovskite/Spiro-OMeTAD/MoO_3_/Au, measured at room temperature. Figure S22: transient absorption spectra and carrier dynamics. Figure S23: transient absorption spectra and carrier dynamics. Table S1: summary of performances of the reported best-performing MAPbI3 PSCs. Table S2: the device parameters of PSCs based on different scanning directions. Table S3: TA decay time (*τ*TA) and the estimated hole transfer efficiency (*η*) from the TA results. Table S4: TA decay time (*τ*TA) and the estimated electron transfer efficiency (*η*) from the TA results.
