# Supplementary material for: Origin of High Efficiency and Long-Term Stability in Ionic Liquid Perovskite Photovoltaic
Source: Research (Wash D C). 2020 Sep 10;2020:2616345. doi: 10.34133/2020/2616345 (PMC7510343; doi:10.34133/2020/2616345)
Supplement: Supplementary Materials — Figure S1: morphology of thin films. Figure S2: scheme image of preparation of the perovskite film. Figure S3: long-term stability of MAAc perovskite films in ambient air for even 2 years under humidity of 30%-80%. Figure S4: thermal (85°C) and light (1-sun AM1.5G illumination) stability of MAAc and DMF perovskite films in an N2-filled glovebox. Figure S5: TGA spectra of MAAc and DMF perovskite powder. Figure S6: air stability of PSCs. Figure S7: light soaking stability of PSCs. Figure S8: the experimental setup for in situ GIWAXS characterization. Figure S9: a transparent oily-like noncrystalline MAAc perovskite film. Figure S10: time evolutions of peak positions for the (110) crystal plane. Figure S11: time evolutions of the full width at half maximum (FWHM) for the (110) crystal plane through multipeak mode fitting. Figure S12: time evolutions of peak areas for the (110) crystal plane through multipeak mode fitting. Figure S13: the azimuthal distribution mapping by radial integration of the (110) diffraction rings of MAAc and DMF perovskite films. Figure S14: the azimuthal distribution mapping by radial integration of the (110) diffraction rings of time evolution of MAAc and DMF perovskite films. Figure S15: FTIR spectra of MAAc-based perovskite powder, MAAc+PbI2 powder from PbI2@MAAc solution, and pure MAAc solvent. Figure S16: the X-ray photoelectron spectroscopy (XPS) spectra of Pb (4f) of MAAc- and DMF-based PbI2 films. Figure S17: Raman spectra of the MAAc-based PbI2 film. Figure S18: the bulk trap state density of MAAc and DMF perovskite films calculated from TA spectra. Figure S19: defect state density of MAAc perovskite, measured by capacitance-voltage (C-V) with the device structure of ITO/SnO2/perovskite/Spiro-OMeTAD/MoO3/Au. Figure S20: the dark current density of the MAAc and DMF perovskite devices with the structure of ITO/SnO2/perovskite/Spiro-OMeTAD/MoO3/Au. Figure S21: admittance spectra of MAAc and DMF perovskite devices with the structure of ITO [file 2616345.f1.docx]

**Supplementary Material**

**Origin of high efficiency and long-term stability in ionic liquid perovskite photovoltaic**

Lingfeng Chao^1^, Tingting Niu^1^, Hao Gu^2^, Yingguo Yang^3^, Qi Wei^1^, Yingdong Xia^2^, Wei Hui^2^, Shouwei Zuo^4^, Zhaohua Zhu^2^, Chengjie Pei^2^, Xiaodong, Li^5^. Jing Zhang^4^, Junfeng Fang^5^, Guichuan Xing^6^, Hai Li^2^, Xiao Huang^2^, Xingyu Gao^3^, Chenxin Ran^1^, Lin Song^1^, Li Fu^1^, Yonghua Chen^1,2*^, Wei Huang^1,2,7*^

^1^Frontiers Science Center for Flexible Electronics, Xi’an Institute of Flexible Electronics (IFE) and Xi’an Institute of Biomedical Materials & Engineering, Northwestern Polytechnical University, 127 West Youyi Road, Xi'an 710072, China.

^2^Key Laboratory of Flexible Electronics (KLOFE) & Institution of Advanced Materials (IAM), Nanjing Tech University (Nanjing Tech), Nanjing 211816, Jiangsu, China.

^3^Shanghai Synchrotron Radiation Facility, Shanghai Institute of Applied Physics, Chinese Academy of Sciences, Shanghai 201204, P. R. China.

^4^Beijing Synchrotron Radiation Facility, Institute of High Energy Physics, Chinese Academy of Sciences, Beijing 100049, P. R. China.

^5^School of Physics and Electronic Science, Ministry of Education, Nanophotonics &Advanced Instrument Engineering Research Center, East China Normal University, Shanghai, 200062 P. R. China.

^6^Institute of Applied Physics and Materials Engineering, University of Macau, Macau, Macao SAR 999078, China.

^7^Key Laboratory for Organic Electronics & Information Displays (KLOEID), and Institute of Advanced Materials (IAM), Nanjing University of Posts and Telecommunications, Nanjing 210023, Jiangsu, China.

These authors contributed equally: Lingfeng Chao, Tinting Niu, Hao Gu, and Yingguo Yang

*Email: [iamyhchen@njtech.edu.cn](mailto:iamyhchen@njtech.edu.cn) and [iamwhuang@nwpu.edu.cn](mailto:iamwhuang@nwpu.edu.cn)

**Supplementary Method**

**Ions conductivity measurement:** The measurement is conducted in air by applying a constant current on Au/Perovskite/Au device (device area: 150 μm × 1000 μm). The voltage response with time is recorded using Keithley 4200-SCS.

**Carrier diffusion and extraction:**

The charge extraction efficiencies (*η*_ce_) of the perovskite/ETL(HTL) films can be estimated according to the following equations^1^:

$k_{CT}= \frac{1}{\tau_{hetero}}-\frac{1}{\tau_{pero}}$ (1)

$\eta_{ce}= \frac{\tau_{hetero}}{\tau_{hetero}+\tau_{pero}}$ (2)

where *k*_CT_ is the charge-carrier transfer rate at the interface, *k*_pero_ is the charge-carrier diffusion rate within the bare perovskite, *τ*_hetero_ is the average PL lifetime of the charge-transport layer/perovskite film, and *τ*_pero_ is the average PL lifetime of the bare perovskite. Furthermore, we calculated the charge carrier *L*_D_ in films using 1D diffusion equation:

$L_{D}= \frac{2d}{\pi}\sqrt{2(\frac{\tau}{\tau_{hetero}}-1)}$ (3)

where *d* is the thickness of the perovskite film.

**Trap Density:**

Under low fluence fs laser pulse excitation (where Auger recombination is negligible) and the assumption that trap states recombination is much slower than band edge radiative recombination. The initial photogenerated charge carrier density can be obtained as^2^:

$n_{c}=\sum_{i} n_{TP}^{i}\left( 0 \right)\left( 1-e^{-\frac{a_{i}\tau_{0}I_{PL}}{k}} \right)+I_{PL}/k$ (4)

Here, $n_{TP}^{i}(t)$is the trap states density and *a_i_* is the product of the trapping cross section and the carrier velocity, *k* is a constant, Fitting the experimental result with equation yields two types of traps in these perovskite thin films, with the bulk (surface/interfacial) traps exhibiting fast (slow) trapping times.

**Carrier Lifetime:**

The kinetics extracted from TA spectra are fitted with multi-exponential decay function^3^:

$I\left( t \right)=1/2\sum_{i=1}^{N} H_{i}\left( t \right)A_{i}\exp\left( -\frac{t}{\tau_{i}} \right)$ (5)

where *t* is the probe time delay, *H_i_* (*t*) = [1+erf(*-t/r-r/2**τ_i_*)] is the rising function, *r* ( ̴ 0.1 ps) is the Gaussian laser pulse width, *A_i_* is the amplitude or pre-exponential function, and *τ_i_* is the decay time.


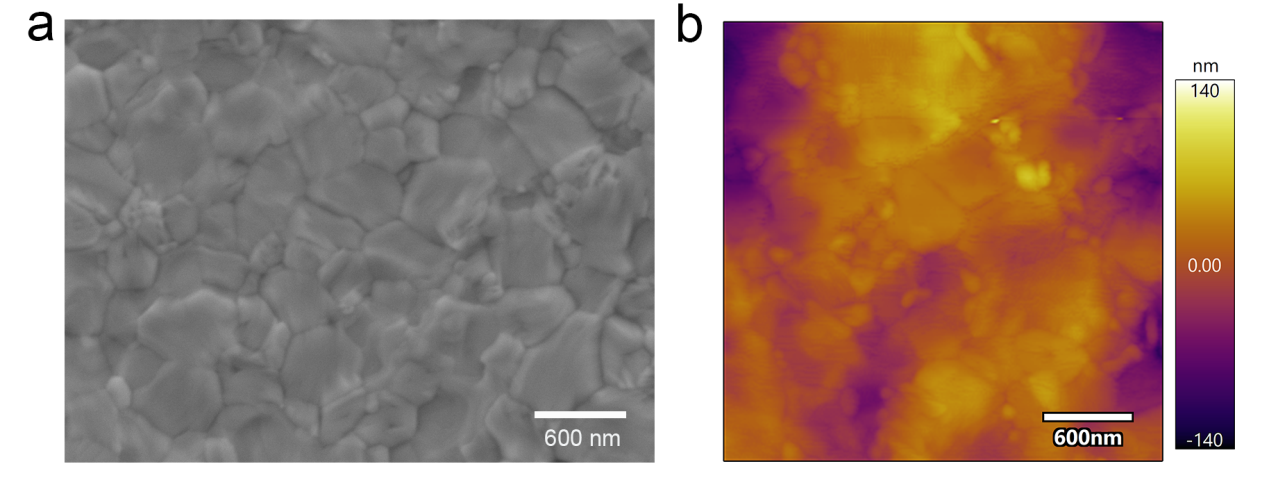


**Figure S1.** **Morphology of thin films**. **a.** The top-view SEM image showing smooth and pinhole-free perovskite film with large grains. **b.** AFM of perovskite film showing low surface roughness (RMS=30.35 nm). The scale bar is 600 nm.


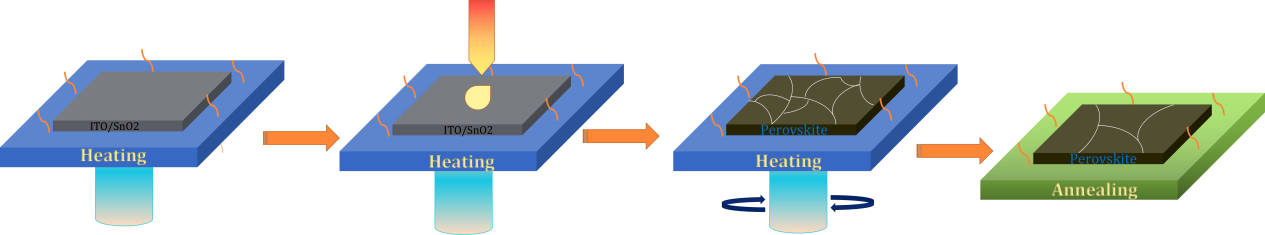


**Figure S2.** **Scheme image of preparation of perovskite film**. The perovskite films are prepared in a natural environment with relative humidity of 20%~80% by one-step spin coating. In the process of preparing the film, the substrate first needs to be preheated for 5 min. The perovskite precursor solution is then dropped on the substrate and spin-coated (4000 rpm) at a constant substrate temperature (90 °C). After spin coating, the film is finally annealed on a hot plate (100 °C).


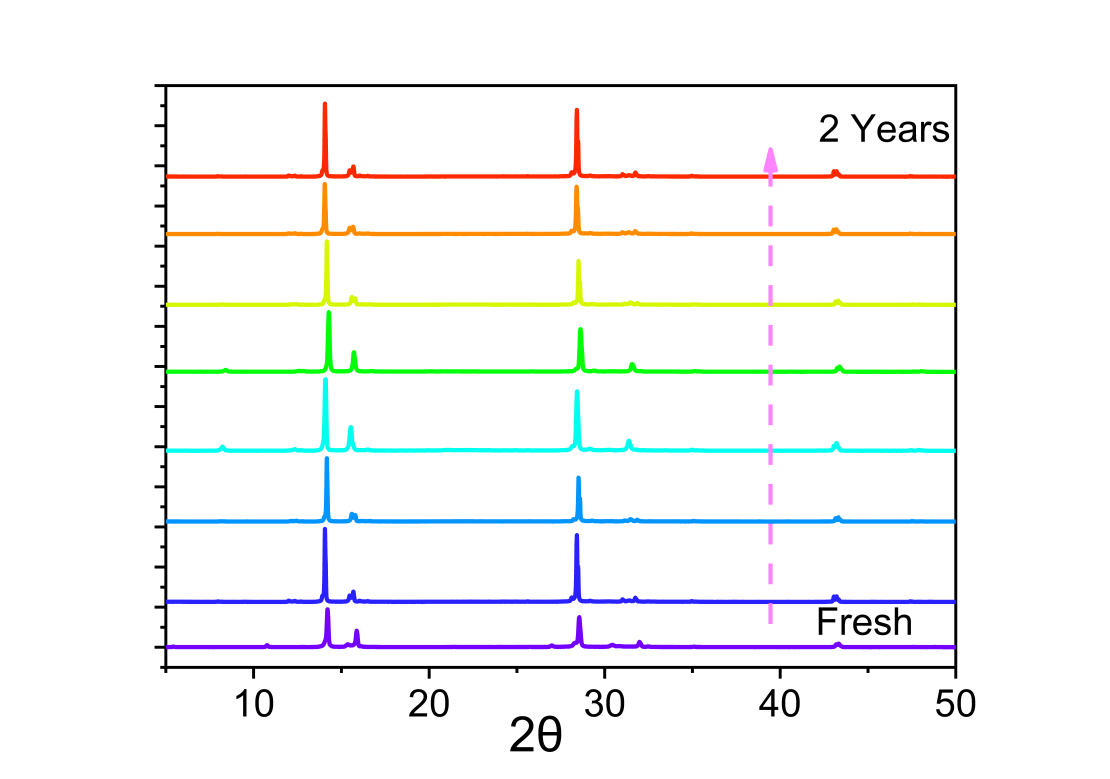


**Figure S3.** Long-term stability of MAAc perovskite films in ambient air for even 2 years under humidity of 30%-80%. The film is placed in a light-proof box and placed in a storage cabinet under natural environment.


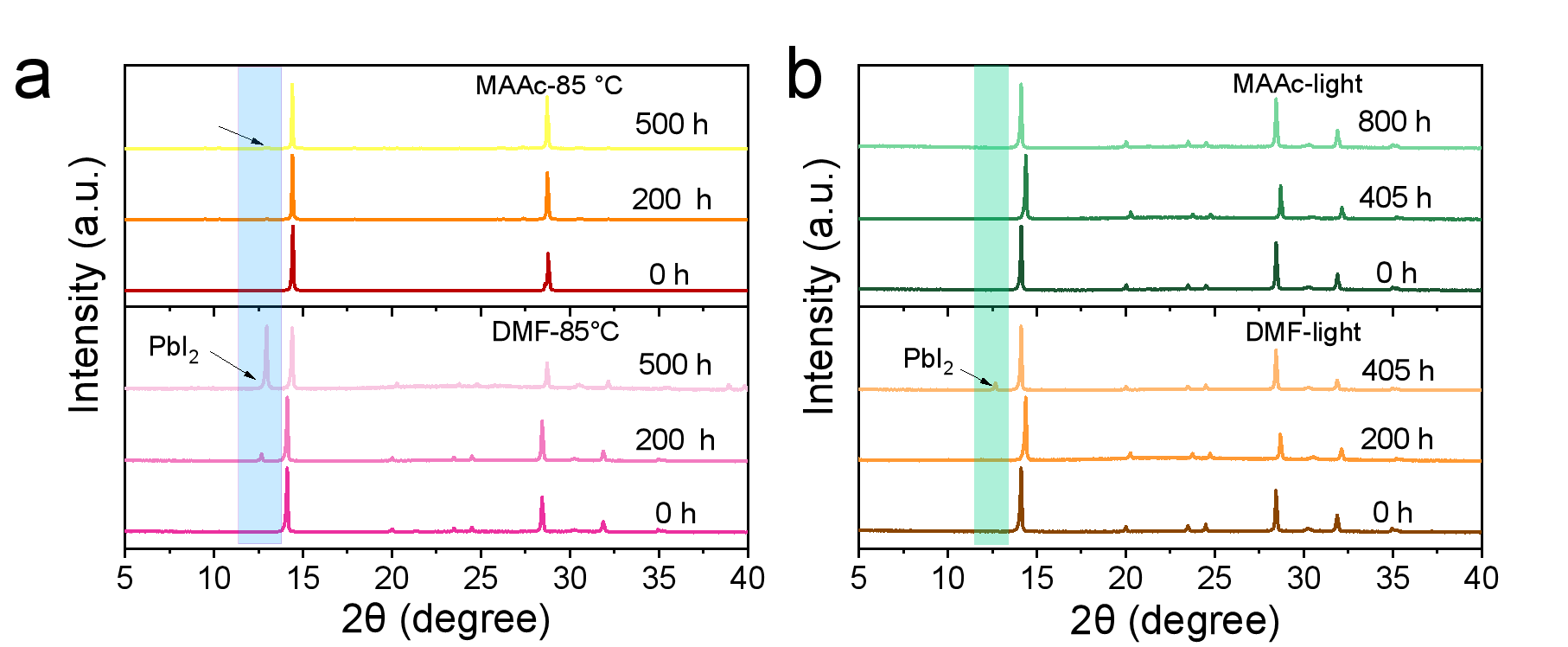


**Figure S4.** Thermal (85 °C) and light (1 sun, AM 1.5G illumination) stability of MAAc and DMF perovskite films in a N_2_-filled glovebox.


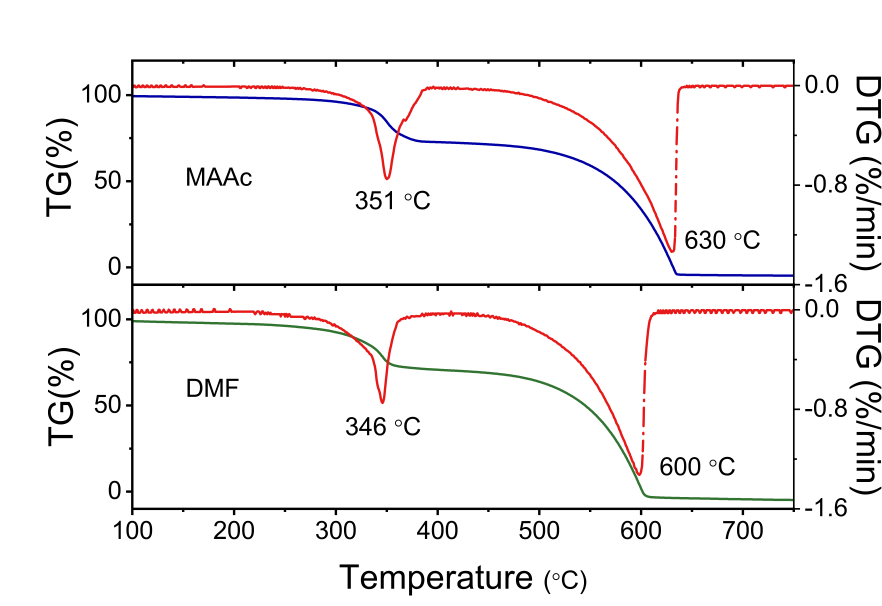


**Figure S5.** TGA spectra of MAAc and DMF perovskite powder. Thermal decomposition profiles of samples were recorded by a thermogravimetric analyzer TA Q500 with a flow rate of 20 ml min^-1^. The temperature varied from 25 to 800 °C at a heating rate of 10 °C min^-1^.


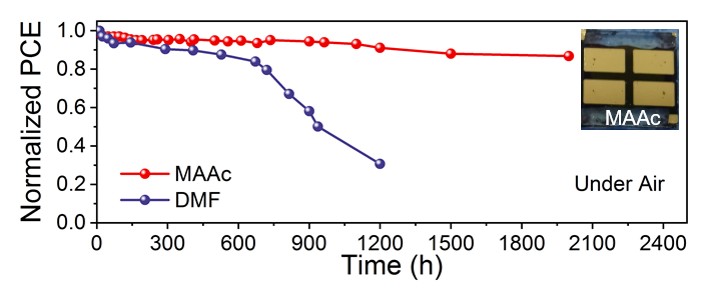


**Figure S6.** Long-term stability. Air stability of non-encapsulated PSCs. The devices are kept in air (relative humidity: 60–80%) and measured regularly in glovebox. The illustration shows the device after being placed in the air for 2500 h

**
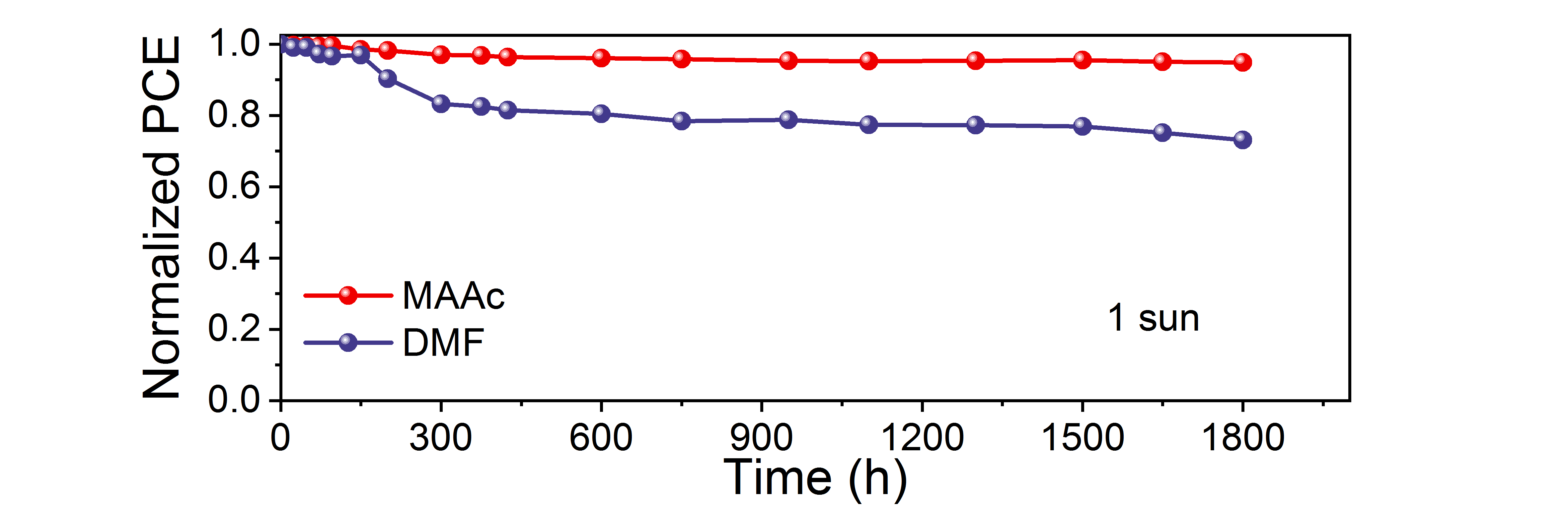
**

**Figure S7.** Long-term stability. Stability of PSCs under continuous 1 sun illumination. The devices are kept and measured regularly in glovebox ﬁlled with N_2_.


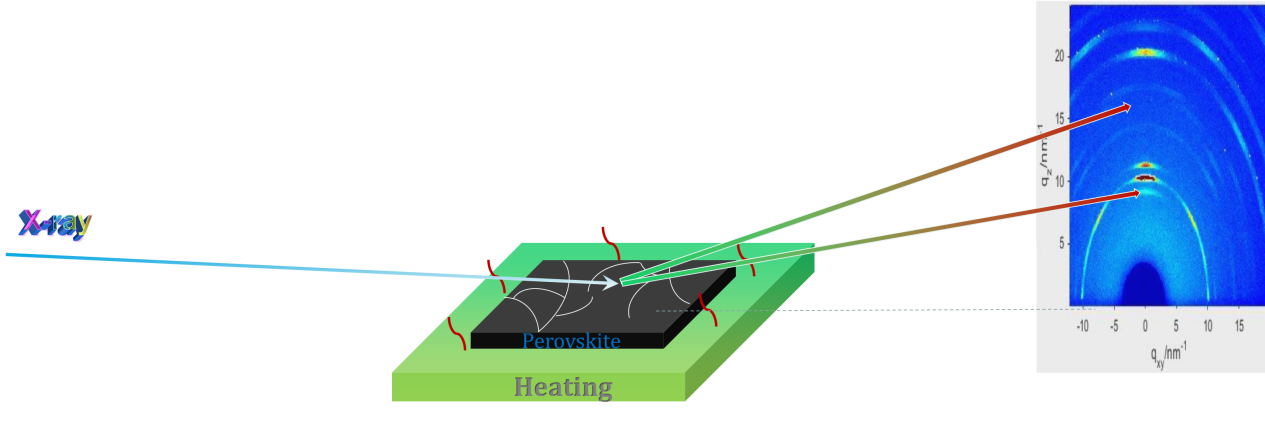


**Figure S8.** **The experimental setup for in situ GIWAXS characterization.** The scheme image of annealing combined with GIWAXS set-up. GIWAXS measurements were conducted at the BL14B1 beamline of Shanghai Synchrotron Radiation Facility (SSRF). the scattering signals were collected by a MarCCD detector. The sample to detector distance was ≈ 223 mm, calibrated with a lanthanum hexaboride (LaB6) sample. After placing the spin-coated film at room temperature on the sample stage at room temperature, the GIWAXS measurement is triggered and the temperature of the sample stage is raised simultaneously.

**
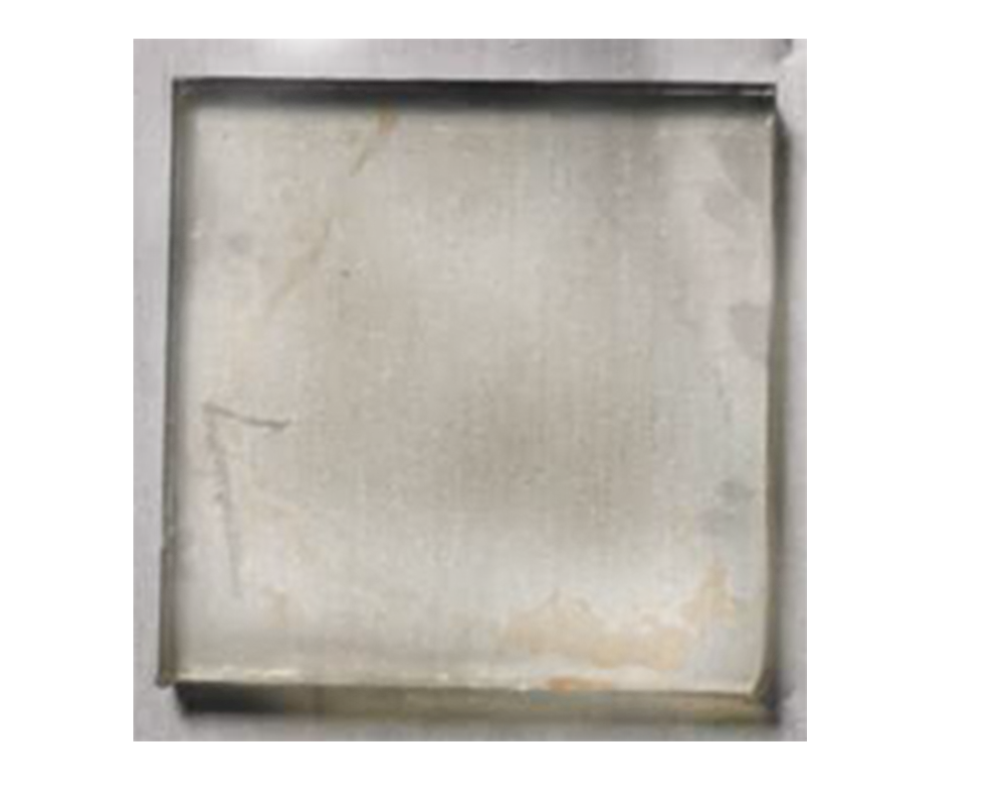
**

**Figure S9.** A transparent oily-like non-crystalline MAAc perovskite film. The MAAc perovskite precursor solution is dropped onto a ITO/SnO_2_ substrate at room temperature during the solution processing. A transparent oily-like non-crystalline perovskite film is obtained after spin-coating.


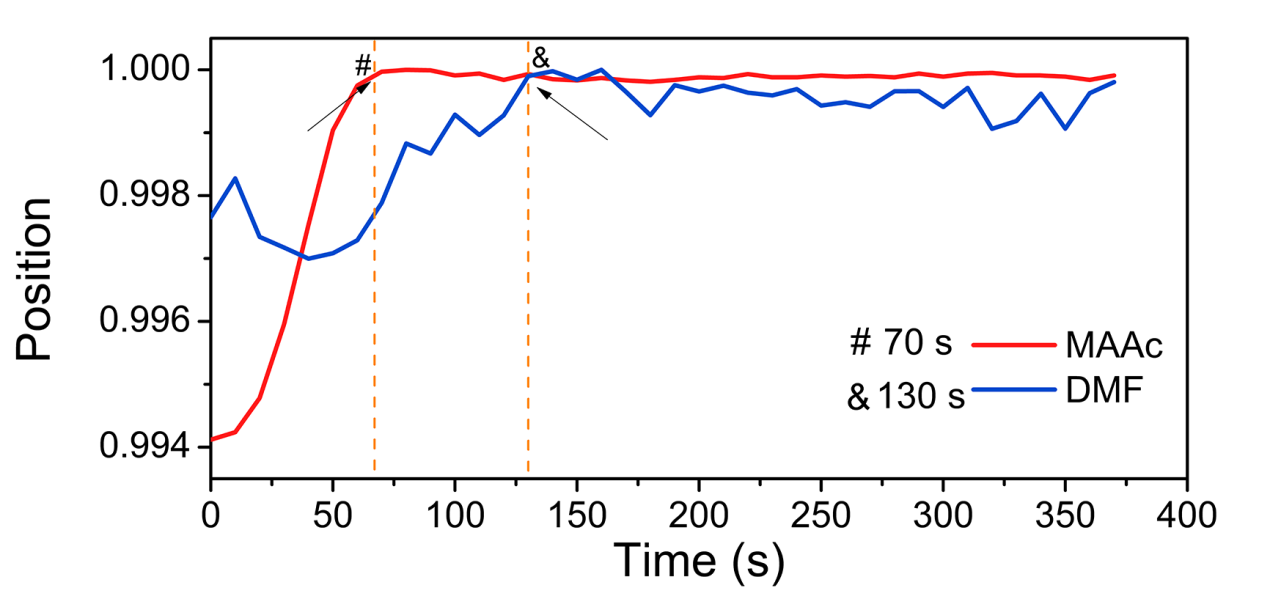


**Figure S10.** Time evolutions of peak positions for (110) crystal plane. Gaussian-function fitting was applied to determine the peak positionof the (110) crysta plane peak through Igor software.


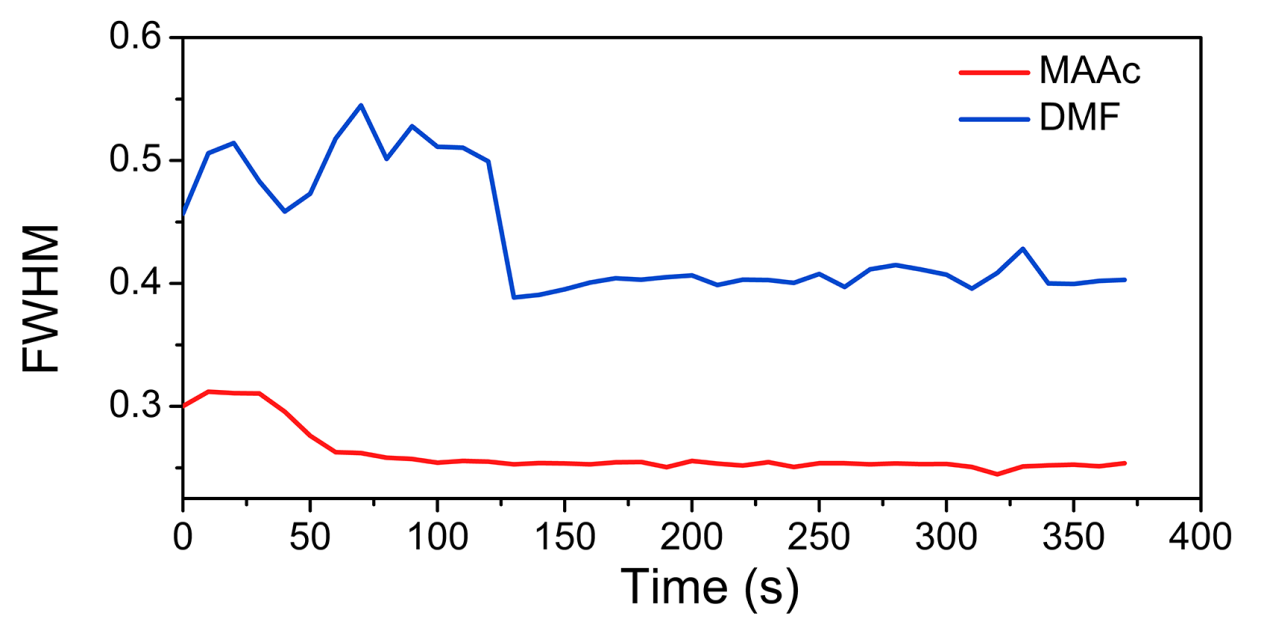


**Figure S11.** Time evolutions of the full width at half maximum (FWHM) for (110) crystal plane through multipeak mode fitting.


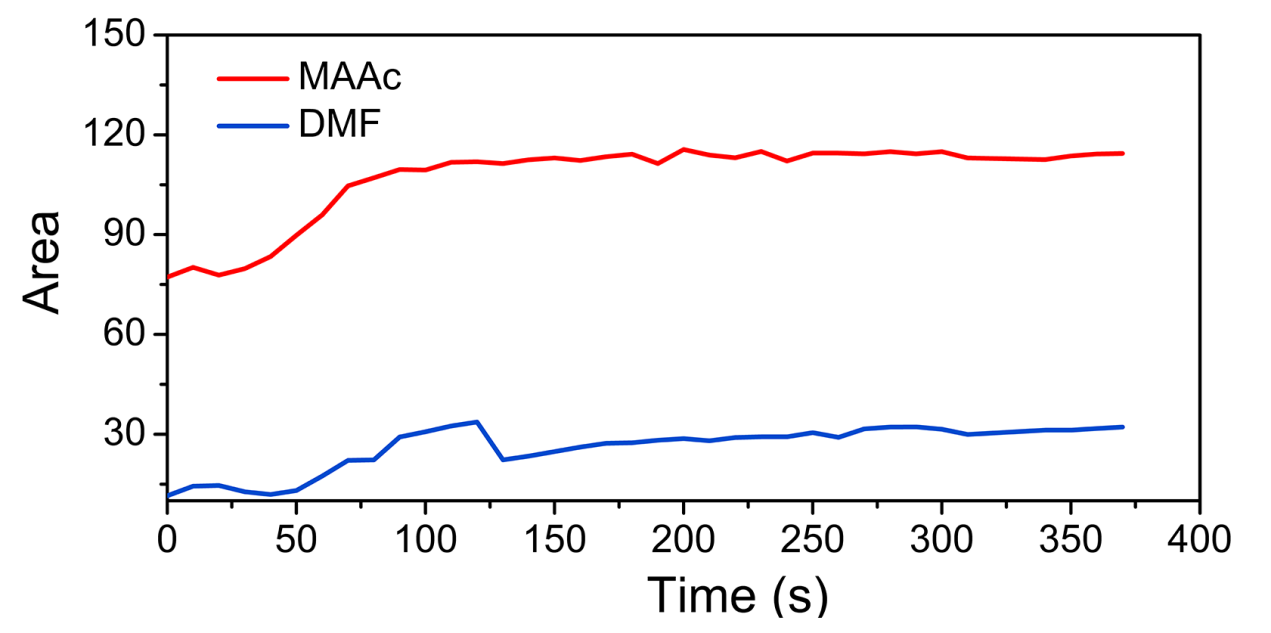


**Figure S12.** Time evolutions of peak areas for (110) crystal plane through multipeak mode fitting.


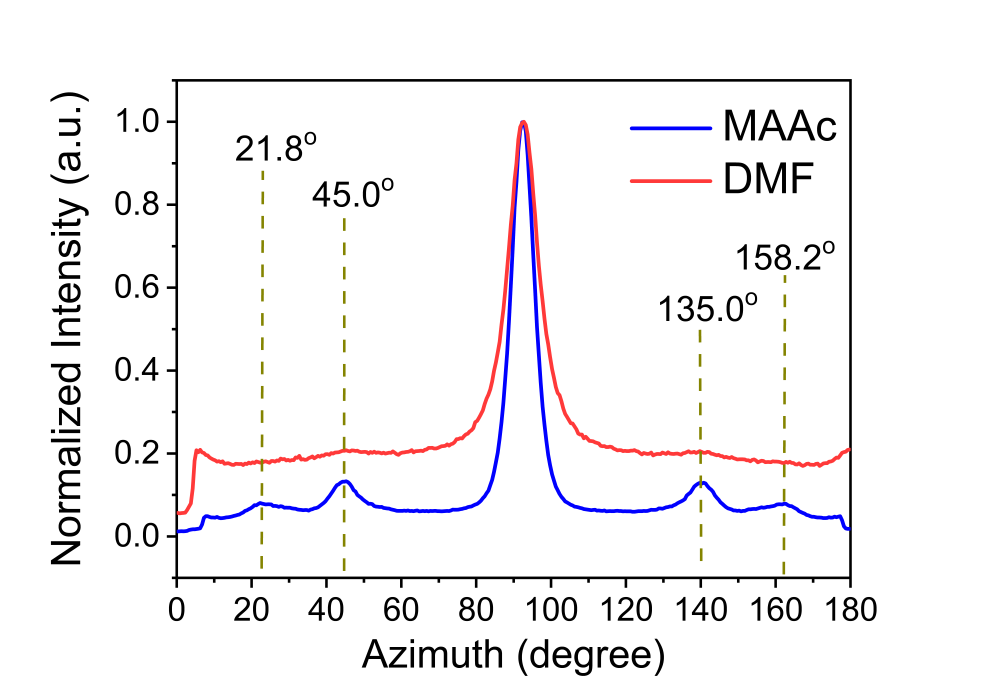


**Figure S13.** The azimuthal distribution mapping by radial integration of the (110) diffraction rings of MAAc and DMF perovskite films.


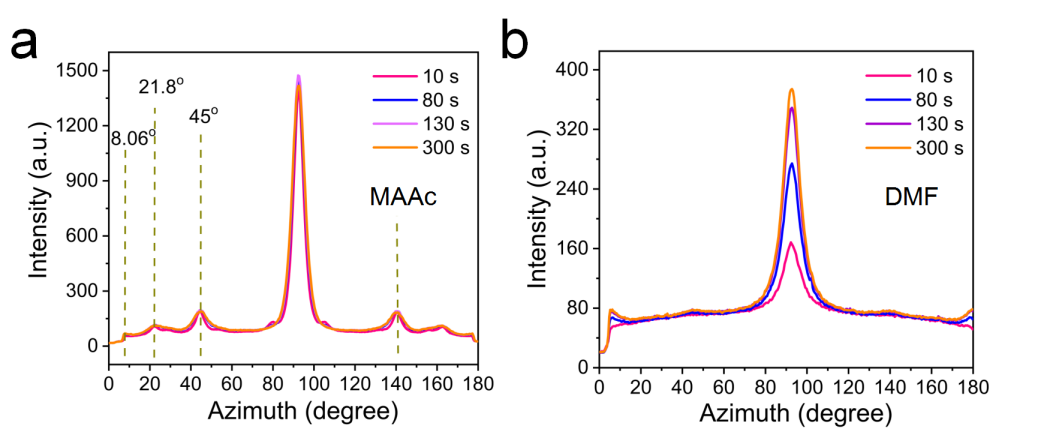


**Figure S14.** The azimuthal distribution mapping by radial integration of the (110) diffraction rings of time evolution of MAAc and DMF perovskite films. a, MAAc. b, DMF.


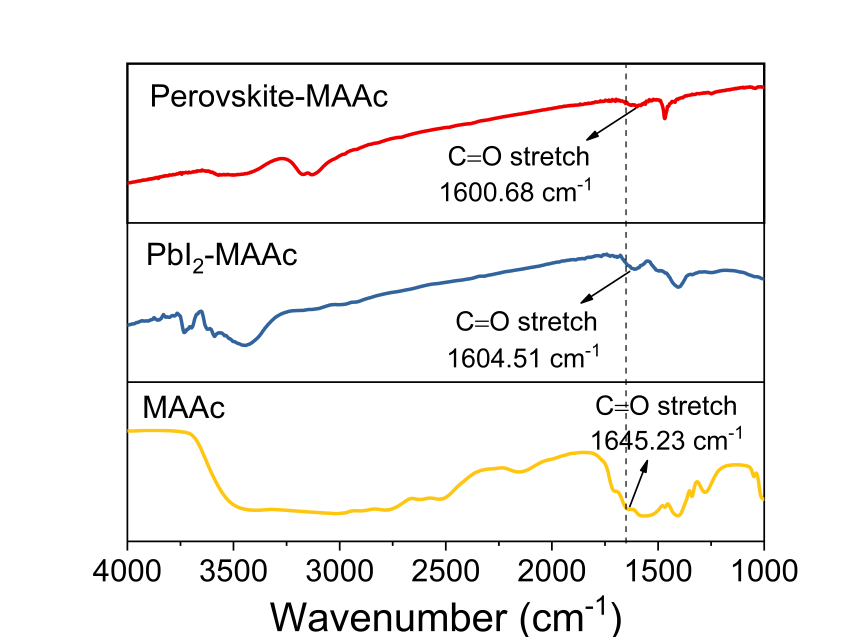


**Figure S15.** FTIR spectra of MAAc based perovskite powder, MAAc+PbI_2_ powder from PbI_2_@MAAc solution and pure MAAc solvent.

**Figure S16.** The X-ray photoelectron spectroscopy (XPS) spectra of Pb (4f ) of MAAc and DMF based PbI_2_ film.


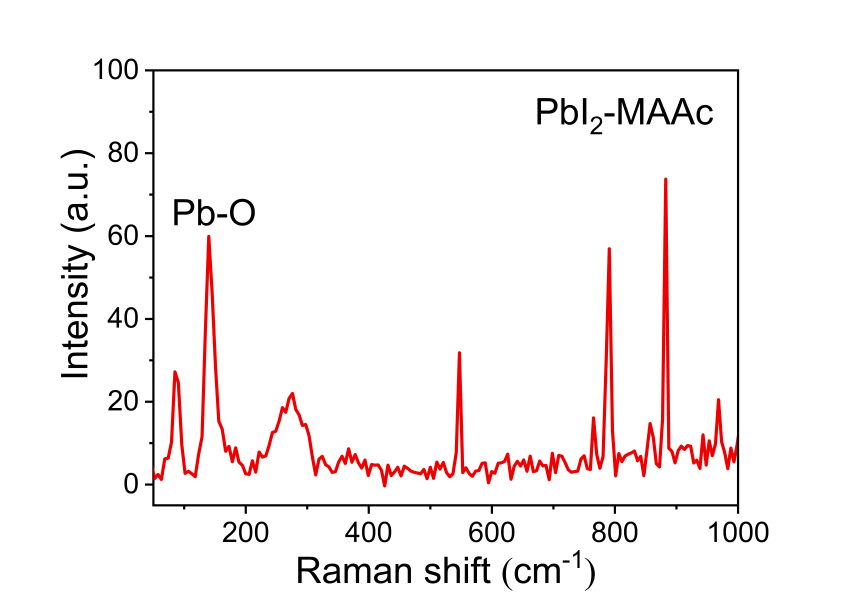


**Figure S17.** Raman spectra of MAAc based PbI_2_ film.

**
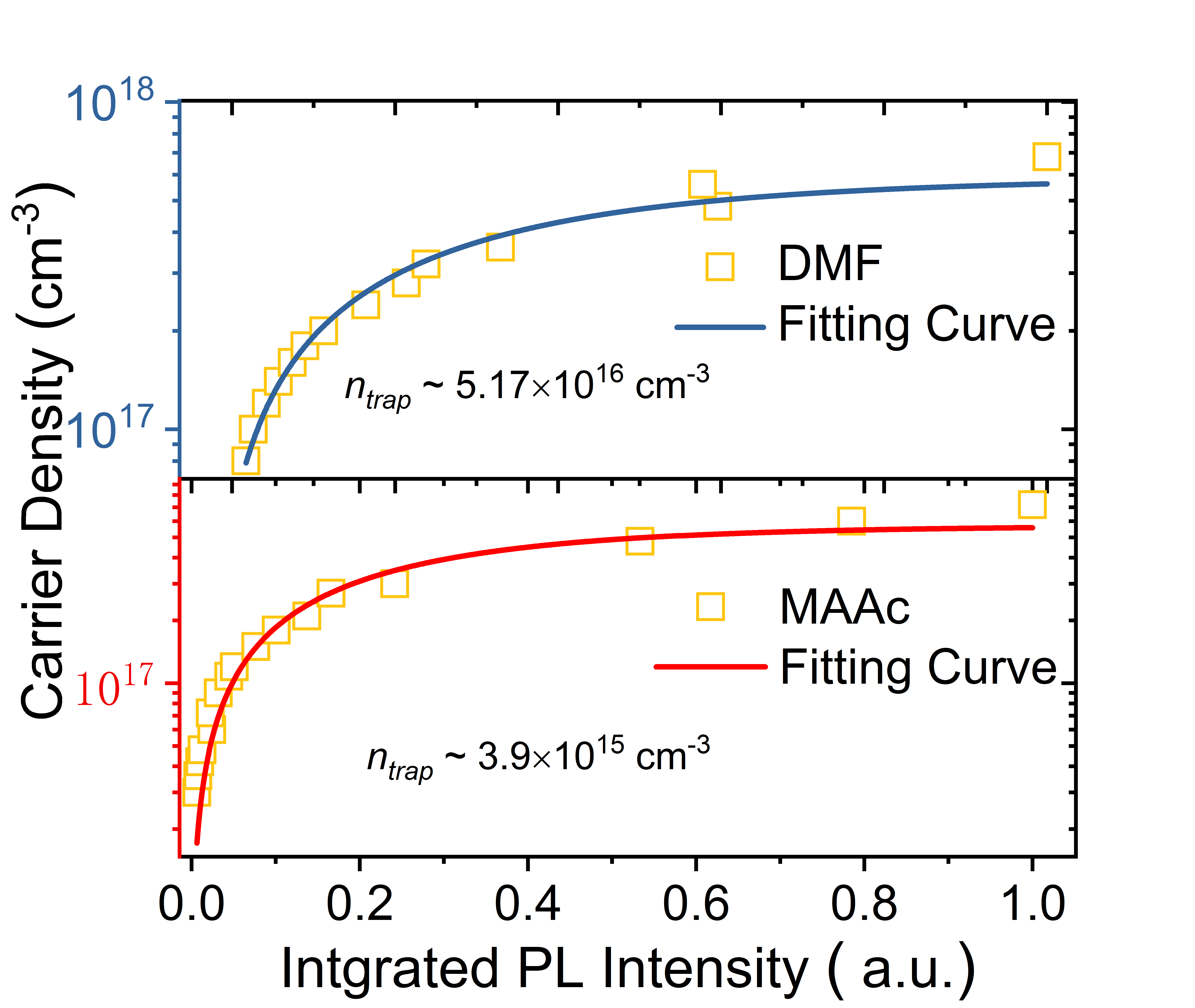
**

**Figure S18.** The bulk trap state density of MAAc and DMF perovskite films calculated from TA spectra.


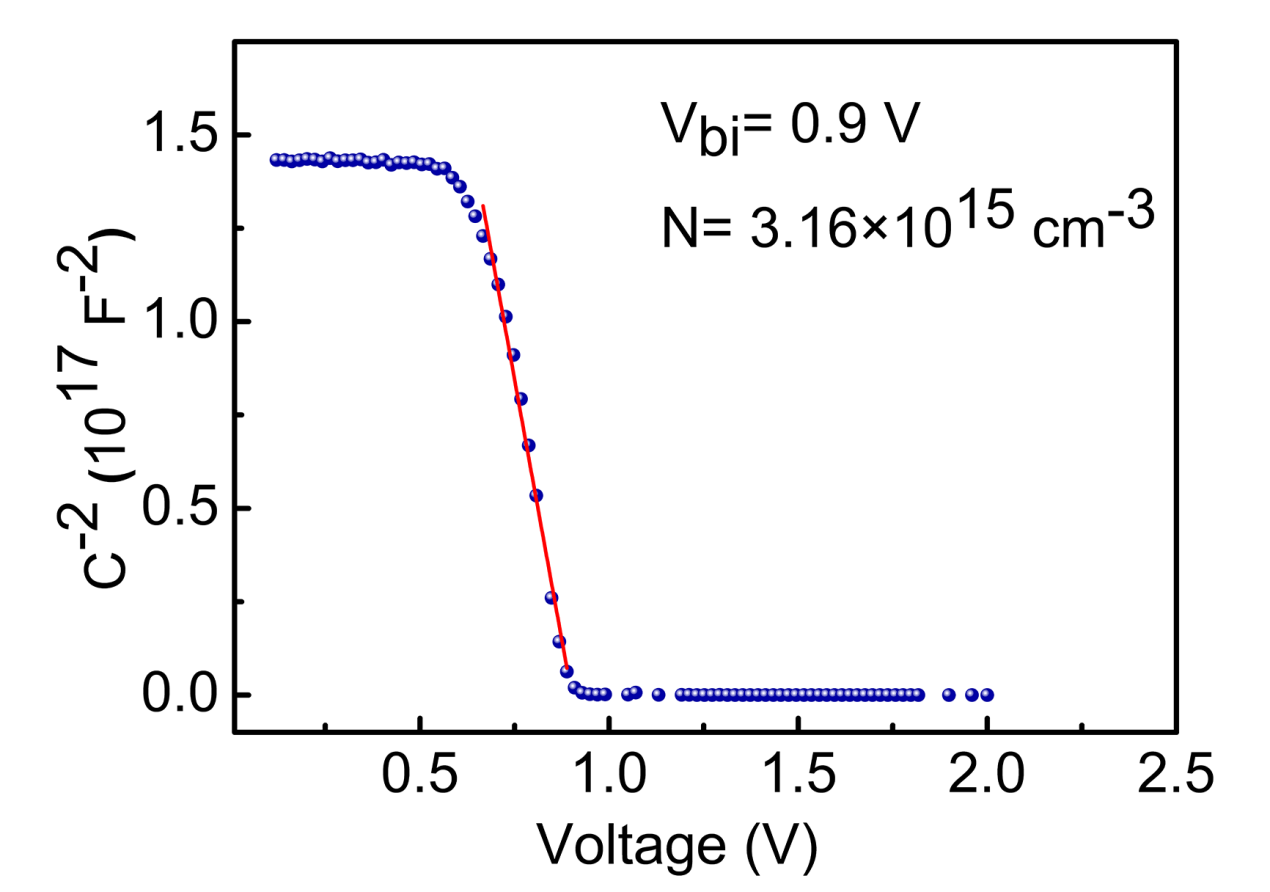


**Figure S19.** Defect state density of MAAc perovskite, measured by capacitance-voltage (C-V) with the device structure of ITO/SnO_2_/perovskite/Spiro-OMeTAD/MoO_3_/Au. Mott-Schottky analysis at 1 kHz for the devices. The distribution of trap state density can be derived from the equation, $N_{T}(E_{\omega})=-\frac{V_{bi}dC}{qWd\omega}\frac{\omega}{kT}$ where V_bi_ is the built-in potential, W is the depletion width, C is the capacitance, o is the applied frequency and T is the temperature. V_bi_ and W are extracted from the C-V measurements^4^.


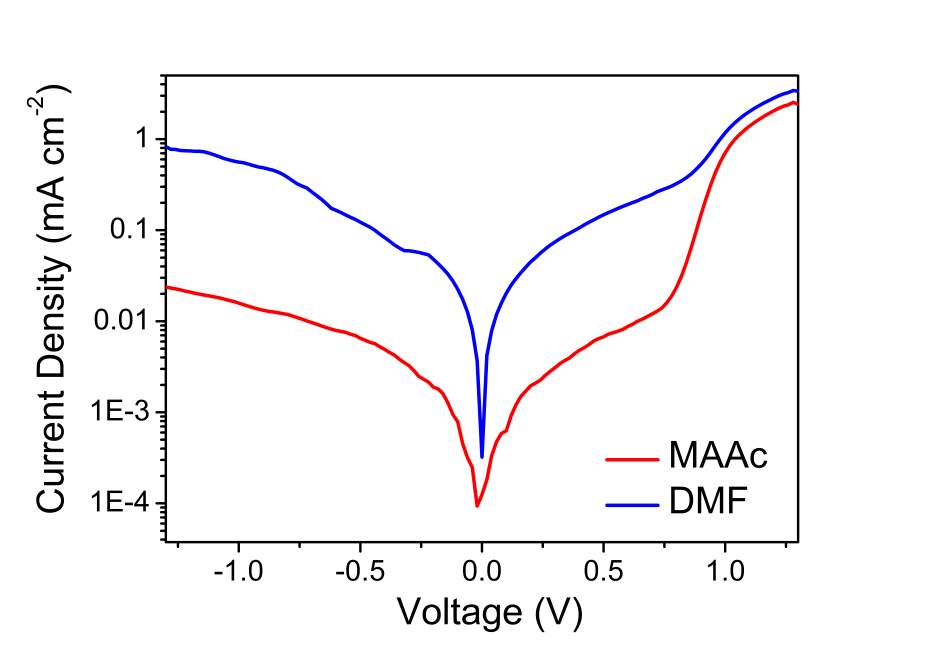


**Figure S20.** The dark current density of the MAAc and DMF perovskite devices with the structure of ITO/SnO_2_/perovskite/Spiro-OMeTAD/MoO_3_/Au.


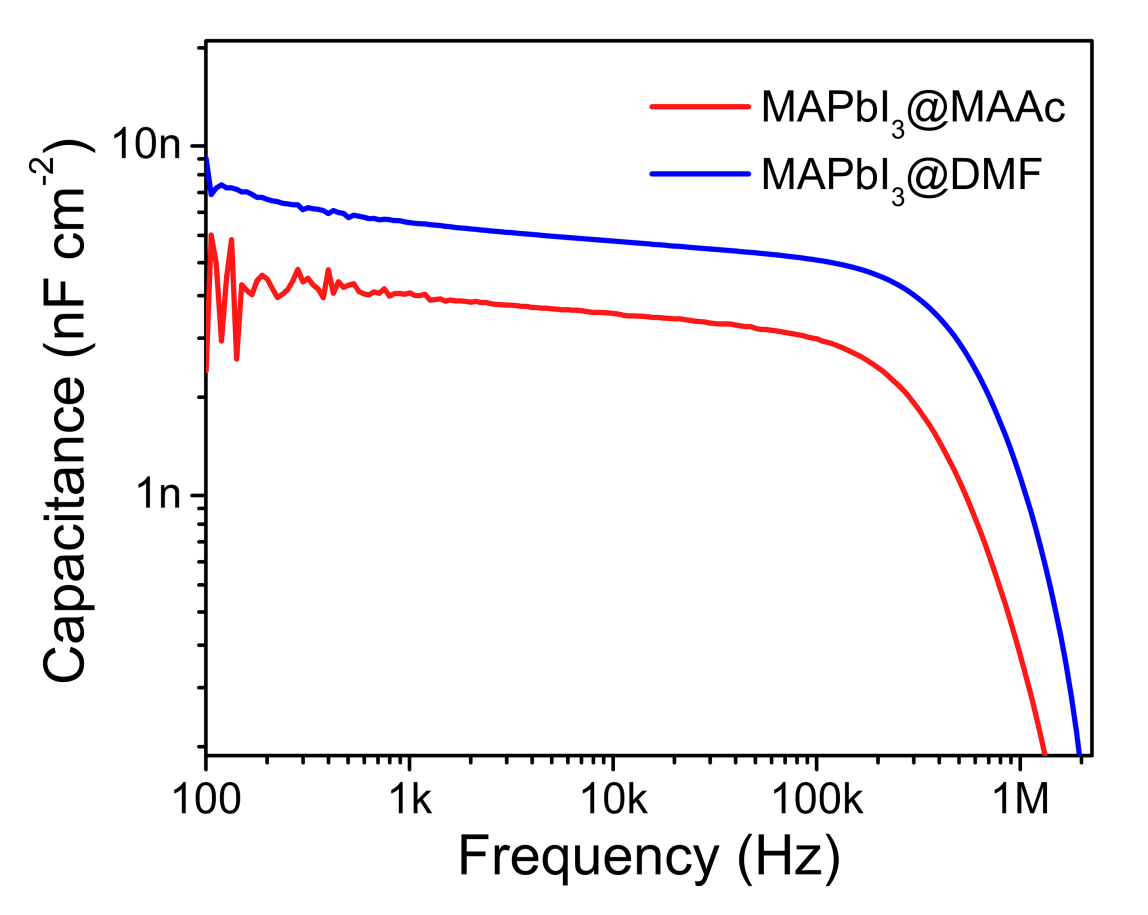


**Figure S21.** Admittance spectra of MAAc and DMF perovskite devices with the structure of ITO/SnO_2_/perovskite/Spiro-OMeTAD/MoO_3_/Au, measured at room temperature.


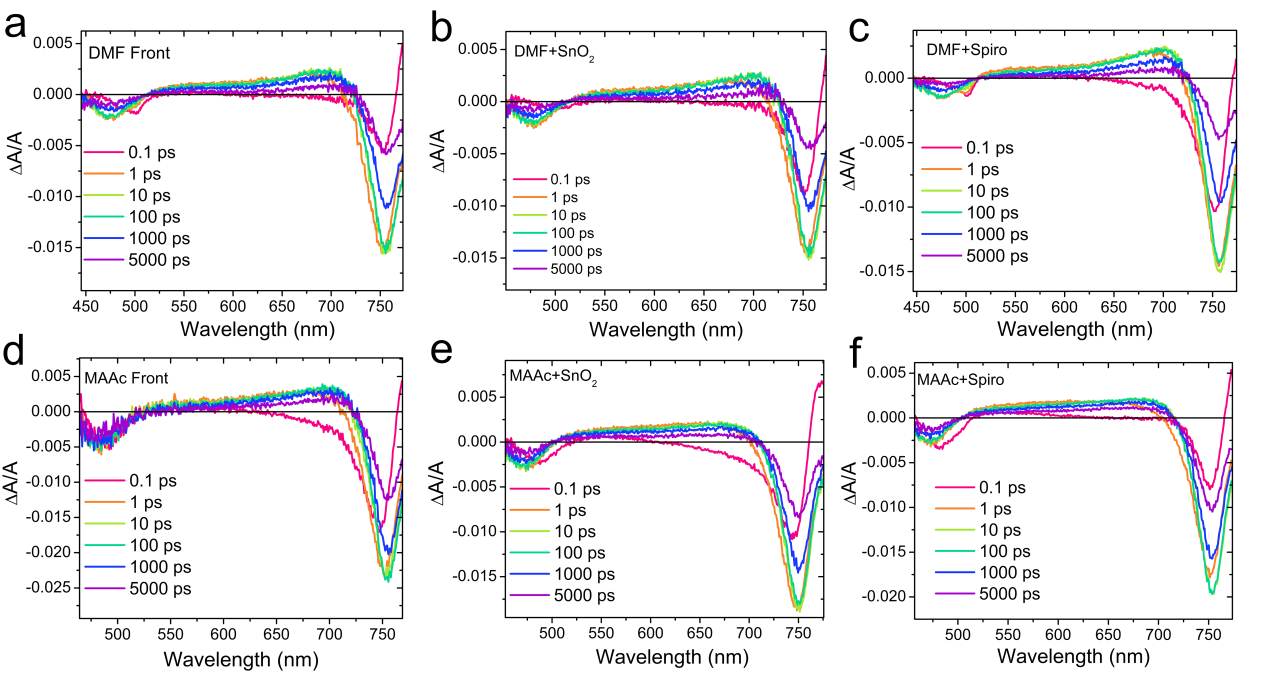


**Figure S22.** **Transient absorption spectra and carrier dynamics.** Representative TA spectra of (a) DMF, (b) DMF/SnO_2_ and (c) DMF/Spiro thin films in response to 400 nm excitation with a fixed excitation density of 1.6 mJ cm^-2^. Representative TA spectra of (d) MAAc, (e) MAAc/SnO_2_ and (f) MAAc/Spiro thin films in response to 400 nm excitation with a fixed excitation density of 1.6 mJ cm^-2^. The thickness of both films is 100 nm.


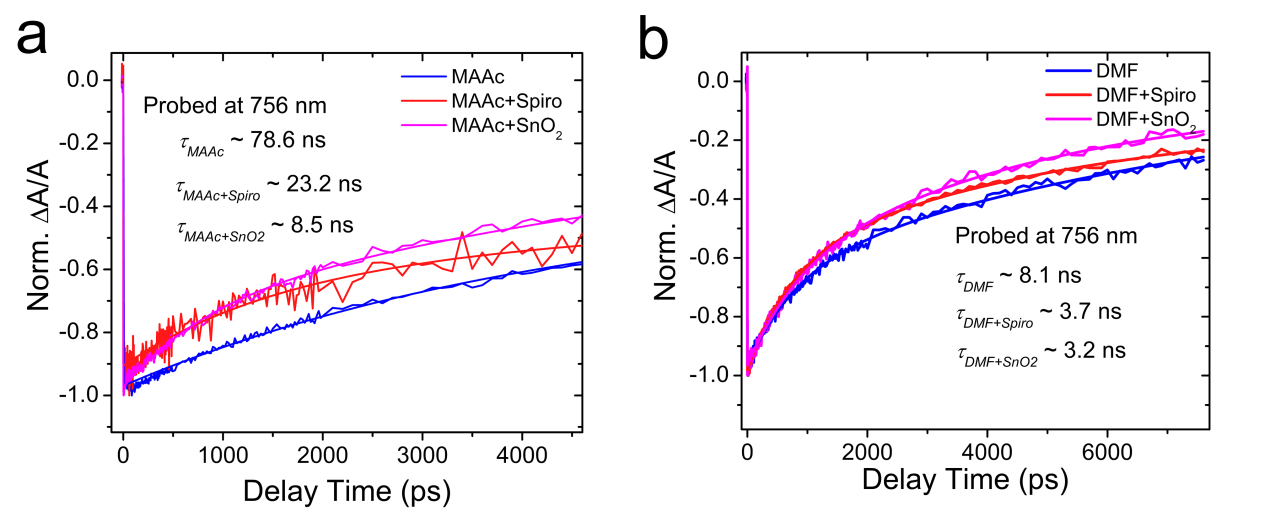


**Figure S23.** **Transient absorption spectra and carrier dynamics.** (a) Kinetic traces of MAAc perovskite film, MAAc/SnO_2_ and MAAc/Spiro thin films probed at band-edge of perovskites. (b) Kinetic traces of DMF perovskite film, DMF/SnO_2_ and DMF/Spiro thin films probed at band-edge of perovskites.

**Table S1. Summary of performances of the reported best-performing MAPbI_3_ PSCs.**

| **Perovskite** | **Device Configuration** | **Jsc**  **(mA cm^-2^)** | | **Voc**  **(V)** | **FF (%)** | **PCE**  **(%)** | **refs** |
| --- | --- | --- | --- | --- | --- | --- | --- |
| MAPbI_3_ | ITO/P3CT-N/perovskite/PCBM/C_60_/BCP/Cu | | 22.80 | 1.11 | 80.20 | 20.22 | 5 |
| MAPbI_3_ | ITO/SnO_2_/perovskite/PTAA/Ag | | 22.97 | 1.14 | 77.13 | 20.25 | 6 |
| MAPbI_3_ | FTO/c-TiO_2_/TiO_2_-Poly(TA)/perovskite+Poly(TA)/PCBM/Au | | 23.48 | 1.14 | 76.00 | 20.40 | 7 |
| MAPbI_3_ | FTO/TiO_2_/perovskite/spiro-OMeTAD/Ag. | | 22.77 | 1.17 | 80.10 | 21.33 | 8 |
| MAPbI_3_ | FTO/c-TiO_2_/m-TiO_2_/perovskite/spiro-OMeTAD/Au. | | 24.27 | 1.10 | 75.00 | 20.02 | 9 |
| MAPbI_3_ | ITO/NiO_x_/perovskite/choline chloride/PCBM/C_60_/BCP/Ag | | 22.00 | 1.06 | 78.00 | 18.20 | 10 |
| MAPbI_3_ | ITO/CPTA/BACl/perovskite/spiro-OMeTAD/MoO_3_/Au. | | 23.16 | 1.11 | 78.01 | 20.05 | 11 |
| MAPbI_3_ | ITO/NiOx/perovskite/PCBM/BCP/Ag | | 22.05 | 1.02 | 81.01 | 18.28 | 12 |
| MAPbI_3_ | ITO/PTAA/MoS_2_/perovskite/PCBM/BCP/Ag | | 22.66 | 1.13 | 80.26 | 20.55 | 13 |
| MAPbI_3_ | ITO/PTAA/perovskite/choline chloride/C_60_/BCP/Cu | | 22.90 | 1.15 | 76.00 | 20.00 | 14 |
| MAPbI_3_ | ITO/PEDOT:PSS/perovskite/C_60_/PCBM/Al | | 22.98 | 1.02 | 77.00 | 18.00 | 15 |
| MAPbI_3_ | ITO/DFH/perovskite/C_60_/PCBM/Ag | | 22.60 | 1.10 | 82.90 | 20.60 | 16 |
| MAPbI_3_ | ITO/PTAA/Perovskite/PCBM/Al | | 23.01 | 1.12 | 81.00 | 20.78 | 17 |
| MAPbI_3_ | FTO/SnO_2_/perovskite/spiro-OMeTAD/Ag | | 22.81 | 1.13 | 77.80 | 20.06 | 18 |
| MAPbI_3_ | ITO/PTAA/ perovskite single crystal/C60/BCP/Cu | | 23.68 | 1.14 | 81.00 | 21.90 | 19 |
| MAPbI_3_ | ITO/SnO_2_/perovskite/spiro-OMeTAD/MoO_3_/Au | | 23.39 | 1.13 | 80.01 | 21.18 | This work |

**Table S2. The device parameters of PSCs based on different scanning directions.**

| Scanning direction | V_OC_ (V) | J_SC_ (mA cm^-2^) | FF (%) | PCE (%) |
| --- | --- | --- | --- | --- |
| Forward | 1.128 | 23.36 | 80.00 | 21.08 |
| Reverse | 1.132 | 23.39 | 80.01 | 21.18 |

**Table S3. TA decay time (τ_TA_) and the estimated hole transfer efficiency (η)** **from the TA results.**

| **Sample** | **τ_perovskite_(ns)** | **τ_Spiro_ (ns)** | **τ_CT_ (ns)** | **η (%)** |
| --- | --- | --- | --- | --- |
| DMF | 8.099 | 3.693 | 6.788 | 54.401 |
| MAAc | 78.621 | 23.197 | 32.906 | 70.495 |

**Table S4. TA decay time (τ_TA_) and the estimated electron transfer efficiency (η) from the TA results.**

| **Sample** | **τ_perovskite_(ns)** | **τ_SnO2_ (ns)** | **τ_CT_ (ns)** | **η (%)** |
| --- | --- | --- | --- | --- |
| DMF | 8.099 | 3.218 | 5.339 | 60.267 |
| MAAc | 78.621 | 8.499 | 9.532 | 89.189 |

**References**

1. D. Xing, N. Mathews, S. Sun et al., Long-range balanced electron- and hole-transport lengths in organic-inorganic CH_3_NH_3_PbI_3_. *Science,* vol. 342, no. 6156, pp. 344-347, 2013.
2. G. Xing, B. Wu, X. Wu, et al., Transcending the slow bimolecular recombination in lead-halide perovskites for electroluminescence. *Nature Communications,* vol. 8, no. 1, article 14558, 2017.
3. D. Giovanni, H. Ma, J. Chua et al., Highly spin-polarized carrier dynamics and ultralarge photoinduced magnetization in CH_3_NH_3_PbI_3_ perovskite thin films. *Nano Letters,* vol. 15, no. 3, pp. 1553-1558, 2015.
4. S. Ye, H. Rao, Z. Zhao et al., A breakthrough efficiency of 19.9% obtained in inverted perovskite solar cells by using an efficient trap state passivator Cu(thiourea)I *Journal of the American Chemical Society,* vol. 139, no. 22, pp. 7504-7512, 2017.
5. X. Li, W. Zhang, Y.-C. Wang, W. Zhang, H.-Q. Wang and J. Fang, In-situ cross-linking strategy for efficient and operationally stable methylammoniun lead iodide solar cells. *Nature Communications,* vol. 9, no. 9, article 3806, 2018.
6. R. Wang, J. Xue, L. Meng, et al., Caffeine improves the performance and thermal stability of perovskite solar cells. *Joule,* vol. 3, no. 6, pp. 1-14, 2019.
7. H. Chen, T. Liu, P. Zhou, et al., Efficient bifacial passivation with crosslinked thioctic acid for high-performance methylammonium lead Iodide perovskite solar cells. *Advanced Materials,* vol. 32, no. 6, article 1905661, 2019.
8. F. Wang, M. Yang, S. Yang, et al., Iodine-assisted antisolvent engineering for stable perovskite solar cells with efficiency >21.3%. *Nano Energy,* vol. 67, no. 1, article 104224, 2020.
9. C. Chen, F. Li, L. Zhu et al., Efficient and stable perovskite solar cells thanks to dual functions of oleyl amine-coated PbSO_4_(PbO)_4_ quantum dots: Defect passivation and moisture/oxygen blocking. *Nano Energy,* vol. 68, no. 6, article 104313, 2020.
10. H. Chen, Q. Wei, M. I. Saidaminov et al., Effcient and stable inverted perovskite solar cells Incorporating secondary amines. *Advanced Materials,* vol. 31, no. 46, article 1903559, 2019.
11. L. Chao, Y. Xia, B. Li, G. Xing, Y. Chen, and W. Huang, Room-temperature molten salt for facile fabrication of efficient and stable perovskite solar cells in ambient air. *Chem,* vol. 5, no. 4, pp. 995-1006, 2019.
12. J. Yang, C. Liu, C. Cai et al., High-performance perovskite solar cells with excellent humidity and thermo-stability via fluorinated perylenediimide. *Advanced Energy Materials,* vol. 9, no. 18, article 1900198, 2019.
13. G. Tang, P. You, Q. Tai et al., *Advanced Materials,* vol. 31, no. 24, article 1807689, 2019.
14. X. Zheng, B. Chen, J. Dai et al., Defect passivation in hybrid perovskite solar cells using quaternary ammonium halide anions and cations. *Nature Energy,*  vol. 2, no. 6, article 17102, 2017.
15. L. Xu, M. Qian, C. Zhang et al., In situ construction of gradient heterojunction using organic VOx precursor for efficient and stable inverted perovskite solar cells. *Nano Energy,* vol. 67, no. 1, article 104244, 2020.
16. Y. Cao, Y. Li, T. Morrissey et a., Dopant-free molecular hole transport material that mediates a 20% power conversion efficiency in a perovskite solar cell. *Energy & Environmental Science,* vol. 12, no. 12, pp. 3502-3507, 2019.
17. K. Wang, L. Zheng, T. Zhu, M. L. Becker and X. Gong, High performance perovskites solar cells by hybrid perovskites co-crystallized with poly(ethylene oxide). *Nano Energy,* vol. 67, no. 1, article 104229, 2020.
18. T.-H. Han, J.-W. Lee, C. Choi et al., Perovskite-polymer composite cross-linker approach for highly-stable and efficient perovskite solar cells. *Nature Communications,* vol. 10, no. 1, article 2019.
19. A. Y. Alsalloum, B. Turedi, X. Zheng, et al., *ACS Energy Letters,* vol. 5, no. 2, article *5*, 657-662, 2020.
